# ILF3 promotes colorectal cancer cell resistance to ferroptosis by enhancing cysteine uptake and GSH synthesis via stabilizing SLC3A2 mRNA

**DOI:** 10.1038/s41419-025-07872-x

**Published:** 2025-07-23

**Authors:** Shuhao Wang, Lu Zhu, Yubing Wang, Yue Han, Qingkun Wang, Wenqing Yang, Lei Zhao, Yu Zhang, Delong Pei, Wankun Huang, Aihua Jin, Zhenhua Lin, Junjie Piao

**Affiliations:** 1 Key Laboratory of Pathobiology (Yanbian University), State Ethnic Affairs Commission, Yanji, China; 2 Department of Urology, Linyi People’s Hosiplital, Linyi, China; 3 Central Laboratory, Yanbian University Hospital, Yanji, China

**Keywords:** Ubiquitylation, Cell death

## Abstract

Ferroptosis, a type of programmed cell death dependent on iron, is characterized by lipid peroxidation of cellular membranes. However, the roles and underlying mechanisms of RNA-binding proteins (RBPs) in modulating ferroptosis in colorectal cancer (CRC) have not been fully explored. In this study, RNA sequencing (RNA-seq) analysis identified ILF3, an RBP, as a crucial regulator of ferroptosis in CRC cells. Our research demonstrated that ILF3 depletion suppressed CRC cell growth and increased sensitivity to ferroptosis. Combined analysis of RNA-seq data and amino acid metabolomics indicated a relationship between ILF3 and glutathione (GSH) synthesis. Further investigation confirmed that ILF3 knockdown reduced GSH synthesis by regulating SLC3A2-mediated cystine uptake. Mechanistically, ILF3 enhances SLC3A2 mRNA stability by interacting with its 3′ UTR, leading to increased cystine uptake. Notably, our observations revealed a frequent increase in ILF3 levels in patients with CRC, which was associated with poor prognosis. The elevated ILF3 expression in CRC appears to be partly due to stimulation by tumor necrosis factor alpha (TNF-α) from the tumor inflammatory microenvironment. Additionally, TNF-α was found to decrease sensitivity to ferroptosis by promoting ILF3 expression. Co-immunoprecipitation and liquid chromatography-mass spectrometry assays revealed that the E3 ligase TRIM17 is involved in TNF-α-induced ILF3 upregulation. Specifically, TNF-α inhibited the interaction between ILF3 and TRIM17, thereby protecting ILF3 from ubiquitin-mediated degradation. This resulted in increased ILF3 levels that counteracted ferroptosis. In summary, our study underscores the oncogenic function of ILF3 in CRC and suggests that ILF3 knockdown may serve as a promising therapeutic approach for CRC.

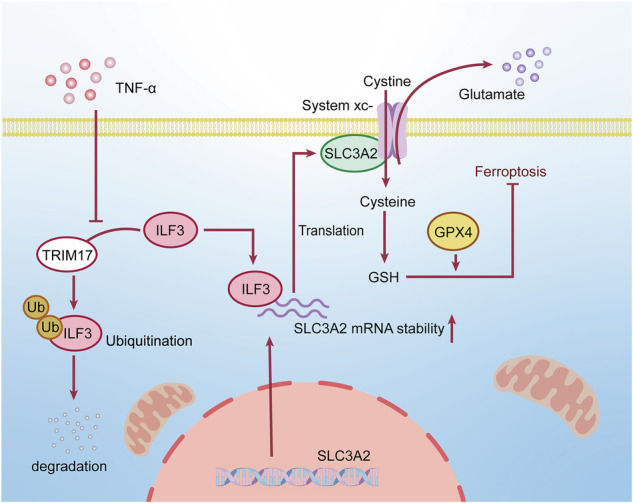

## Introduction

Ferroptosis is a form of programmed cell death driven by lipid peroxidation and dependent on iron [1, 2]. Ample evidence suggests that ferroptosis is inhibited in most tumor cells, and inducing ferroptosis—either alone or in combination with conventional therapies—is a promising antitumor strategy [3, 4]. Therefore, a deeper understanding of ferroptosis may provide effective strategies for cancer therapy.

RNA-binding proteins (RBPs) are a group of proteins that interact specifically with RNA molecules, influencing their fate and function after transcription [5]. Growing evidence indicates that RBPs play essential roles in multiple biological processes in cancer, including proliferation, apoptosis, angiogenesis, senescence, and epithelial–mesenchymal transition [6–8]. Although the role of RBPs in cancer progression is becoming clearer, only a limited number of RBPs have been identified as regulators of ferroptosis. To address this gap, we screened for RBPs that are altered during ferroptosis in colorectal cancer (CRC). Using RNA transcriptome sequencing in RSL3-treated SW620 cells, we identified the top 10 RBPs associated with ferroptosis. Among them, Interleukin enhancer-binding factor 3 (ILF3) emerged as a key candidate, which was further validated by RT-qPCR and western blotting. ILF3, also known as NF90/NF110 [9], belongs to the double-stranded RBP family and plays a crucial role in RNA metabolism, ranging from transcription to degradation [10–12]. ILF3 has been shown to promote angiogenesis by stabilizing VEGF mRNA [13]. YuKi et al. demonstrated through RNA immunoprecipitation (RIP) analysis that NF90 inhibits the translation of endogenous mRNAs containing AU-rich characteristic sequences [14]. Recently, ILF3 was found to directly regulate the stability of SGOC mRNA, thereby upregulating SGOC expression and promoting tumorigenesis [15]. However, the role of ILF3 in modulating ferroptosis in CRC remains unclear.

In this study, we demonstrated that ILF3 expression is elevated in CRC tissues and that high ILF3 expression is associated with poor prognosis. Mechanistically, ILF3 depletion inhibits CRC cell proliferation, cystine uptake, and GSH synthesis by destabilizing SLC3A2 mRNA through its interaction with the 3′ UTR. Additionally, we found that TNF-α promotes CRC cell resistance to ferroptosis by upregulating ILF3. Moreover, TNF-α protects ILF3 from ubiquitination by reducing the interaction between TRIM17 and ILF3. Overall, our study comprehensively illustrated the mechanism by which ILF3 regulates ferroptosis and identifies ILF3 as a potential therapeutic target for CRC.

## Methods

### Cell culture and clinical samples

CRC cell lines, including SW620, SW480, DLD-1, HCT116, RKO, and HEK293T, were obtained from the American Type Culture Collection (ATCC, Rockville, MD, USA). SW620, SW480, DLD-1, and HEK293T cells were grown in DMEM with 10% fetal bovine serum, while HCT116 and RKO cells were maintained in 1640 medium supplemented with 10% fetal bovine serum. All cells were cultured at 37 °C in an atmosphere containing 5% CO_2_. Each cell line underwent routine STR verification. Fourteen pairs of human colorectal cancer specimens and corresponding adjacent non-cancerous tissues used in this research were supplied by Yanbian University Hospital. The study protocol received approval from the Ethics Committee of Yanbian University Hospital.

### Antibodies and reagents

The following antibodies were used in this study: anti-GAPDH(#60004-1-Ig), anti-ACSL4 (#22401-1-AP), anti-BHMT (#15965-1-AP), anti-GPX4 (#14432-1-AP), anti-GNL3 (#15060-1-AP), anti-HA-Tag (#66006-2-Ig), anti-ILF3 (#19887-1-AP), anti-LARP7 (#17067-1-AP), anti-SLC3A2 (#15193-1-AP), anti-TFRC(#17435-1-AP), anti-TRIM17(#13663-1-AP), anti-Ub(#10201-2-AP), anti-SLC7A11/xCT (#26864-1-AP), anti-ZFP36 (#12737-1-AP), anti-NF-κ B p65(#10745-1-AP), anti-Phospho-NF-κ B p65(#82335-1-RR) were purchased from Proteintech; anti-FTH1 (#3998S),anti-DYKDDDDK Tag (#14793 T),anti-His Tag (#12698 T),anti-Myc Tag (#2276S) were purchased from CST; anti-SYNE1 (#ab192234), anti-4-HNE (#ab48506), anti-Ki-67 (# ab16667) were purchased from abcam; anti-3PGDH (#sc-100317), anti-CBS (#sc-133154), anti-GSS (#sc-365863), anti-GCL (#sc-271330), anti-γ-GCSc (#sc-390811) were purchased from Santa Cruz.The following reagents were used in this study: RSL3 (#HY-100218A), Erastin (#HY-15763), Chloroquine (#HY-17589A), Ferrostatin-1 (#HY-100579), 3-Methyladenine (#HY-19312). Necrostatin-1 (#HY-15760), Z-VAD-FMK (#HY-16658B),Cycloheximide (#HY-12320) were purchased from MedChemExpress (MCE); TNF-α (#HZ-1014) was purchased from proteintech; MG132 (#A2585) was purchased from APExBIO.

### Immunohistochemistry (IHC) analysis

Immunohistochemical sections were deparaffinized in an oven, then hydrated, antigenically repaired, and endogenous peroxidase blocker, primary antibody was added dropwise to each slice sequentially and overnight at 4 °C. On the next day, the primary antibody was washed, reaction enhancement solution was added sequentially, the secondary antibody was incubated and freshly prepared DAB chromogenic solution (ZLI-9018, ZSGB-BIO) was added, and the degree of coloration was observed under the microscope, then stained with hematoxylin, dehydrated, and then neutral dendrimer-sealed, and then air-dried to capture the images under the microscope.

### Cystine uptake

SW620 cells and DLD-1 cells were seeded at a density of 1 × 10^4^ cells/well in a black transparent-bottomed 96-well cell culture plate and incubated overnight at 37 °C in an incubator. The next day, the supernatant was removed, and the cells were washed three times with serum-free culture medium without cystine. Add reagents in sequence according to the instructions in the Cystine Assay Kit (UP05-DOJINDO) and measure the fluorescence intensity using a fluorescence microplate reader (Ex/Em = 490/535 nm).

### Co-IP

Lyse the cells with IP lysis buffer (P0013, Beyotime). Centrifuge at 13,000 rpm for 20 min and incubate the supernatant with the antibody in a shaker at 4 °C overnight. The next day, the protein A/G magnetic beads (HY-K0202, MedChemExpress) were washed three times with pre-chilled PBS, and then blocked with 0.2% BSA for 1 h. The magnetic beads were then added to the supernatant and incubated on a shaker at 4 °C for 6 h. The supernatant was removed, and the magnetic beads were washed three times with PBS. Then 40 µL of 2X loading buffer was added and heated in a metal bath at 95 °C for 5 min for protein blotting analysis.

### RNA-binding protein immunoprecipitation (RIP)

RIP experiments were performed using RNA Immunoprecipitation Kit (P0102, Geneseed Biotech) 1 × 10^7^ SW620 cells were collected and lysed with 1 ml BufferA (1*), centrifuged and the supernatant was taken, 100 ul of supernatant was taken as Input control, and the magnetic beads were washed, followed by spinning the reaction of the remaining 900 ul of supernatant with 100 ul of proteinA+G-coupled magnetic beads at 4 °C overnight. On the following day, 1 ml of BufferB (1*) was added to the magnetic bead complexes, and the washing was repeated 6 times, followed by elution of the complexes bound to the magnetic beads for RNA purification by overcolumn. RT-qPCR was used to analyse the RNA products, and the relative expression of the target genes was calculated according to the formula of 2-^ΔΔ^Ct.

### Fluorescence in situ hybridization (FISH) analysis

The expression of SLC3A2 mRNA in CRC tissue microarrays was detected using FISH probe mixtures (Gefanbio, Shanghai, China). The 5’-end of the probes was labeled with Cy3. The SLC3A2 mRNA probes were hybridized with tissue sections overnight at 37 °C. Nuclei were counterstained with DAPI, and images were observed and captured under a fluorescence microscope.

### Xenograft mouse models

Five-week-old female nude mice used in this study were purchased from Beijing Vital River Company. For the ILF3 knockdown and SLC3A2 overexpression groups, SW620 cells transfected with LV-NC, LV-sh-ILF3#1, LV-sh-ILF3#3, or LV-OE-SLC3A2 were suspended in serum-free DMEM, and 5 × 10⁶ tumor cells were injected subcutaneously in a total volume of 100 μL per mouse. Tumor volume and weight were measured every 3 days. Tumor volume was calculated using the formula:

$${\rm{Volume}}={\rm{length}}\times {{\rm{width}}}^{2}\times 1/2$$

For the RSL3 injection group, when tumors reached approximately 100 mm³, mice were randomly divided into four groups: the sh-control (sh-Con) group, thesh-Con+RSL3 group, the sh-ILF3#3 group, and the sh-ILF3#3 + RSL3 group. RSL3 (50 mg/kg) was injected intraperitoneally twice per week. After 2 weeks of consecutive injections, tumors were excised, photographed, weighed, and stored for further analysis.

### Statistical analysis

Statistical analyses were conducted using SPSS version 26.0 (SPSS Inc., USA) and GraphPad Prism version 9.0 (GraphPad Software Inc., USA). Each experiment was replicated a minimum of three times. The data are presented as mean values with standard deviation. Details regarding specific statistical analyses can be found in the legend of each figure. A *p*-value of less than 0.05 was considered indicative of statistical significance (ns: not significant, *P < 0.05, **P < 0.01, ***P < 0.001).

## Results

### ILF3 is decreased in RSL3-mediated ferroptosis

**Fig. 1:**
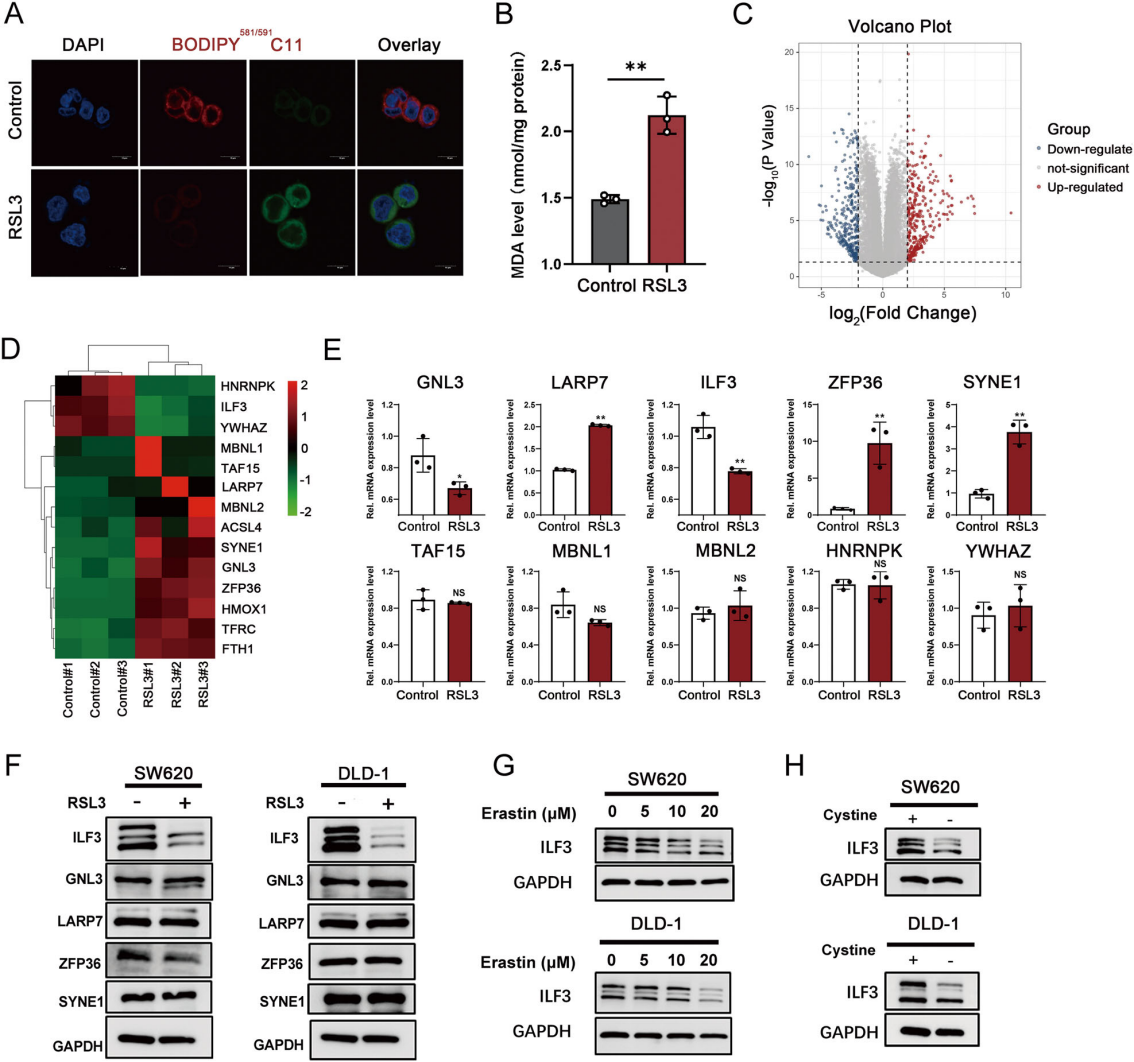
ILF3 is decreased in RSL3-mediated ferroptosis. **A** Lipid peroxidation levels in SW620 cells treated with RSL3 (10 μM, 24 h) were detected using a BODIPY (581/591) C11 probe. Scale bar = 10 μm. Red represents reductive status, green represents oxidative status, and blue represents DAPI staining. **B** MDA levels in SW620 cells treated with RSL3 (10 μM, 24 h) compared to the control group. **C** Volcano plot showing 10,777 DEGs in SW620 cells after RSL3 treatment (10 μM, 24 h) (|log₂FC | ≥ 1, *p*-value ≤ 0.05). **D** Heatmap displaying the top 10 RBPs with significant expression differences and several related positive genes ACSL4, HMOX1, TFRC, and FTH1 in SW620 cells. **E** RT-qPCR analysis of mRNA levels of the top 10 RBPs following RSL3 treatment (10 μM, 24 h). **F** Western blot analysis of ILF3, GNL3, LARP7, ZFP36, and SYNE1 protein levels in SW620 and DLD-1 cells treated with RSL3 (10 μM, 24 h). **G** Western blot analysis of ILF3 protein levels in SW620 and DLD-1 cells treated with various concentrations of Erastin. **H** Western blot analysis of ILF3 protein levels in SW620 and DLD-1 cells subjected to cystine deprivation.

To identify key RBPs involved in ferroptosis regulation in CRC, we constructed a ferroptosis cell model in SW620 cells by treating them with RSL3. Ferroptosis was confirmed by assessing Glutathione Peroxidase 4 (GPX4) expression, malondialdehyde (MDA), and Lipid peroxidation levels (Fig. 1A, B and Figure S1A). Transcriptome sequencing identified 10,777 differentially expressed genes (DEGs) (Fig. 1C), among which the top 10 candidate RBPs were selected for further validation (Fig. 1D). Interestingly, LARP7, SYNE1, and ZFP36 mRNA levels were significantly upregulated after RSL3 treatment, whereas ILF3 and GNL3 were downregulated (Fig. 1E). Western blot analysis further validated that ILF3 was the only RBP significantly downregulated following RSL3 treatment (Fig. 1F). Similar results were observed in another CRC cell line DLD-1(Fig. 1F and Figure S1B–D).

To confirm these findings, we used erastin, another well-established ferroptosis inducer. Consistently, ILF3 expression decreased following erastin-induced ferroptosis (Fig. 1G). Since cystine deprivation is a well-known trigger of ferroptosis [16–18], we examined ILF3 expression under cystine-deprived conditions. Interestingly, ILF3 levels were downregulated in CRC cells upon cystine deprivation (Fig. 1H). These results suggest that ILF3 expression decreases during ferroptosis and may play a crucial role in ferroptosis regulation.

### Knockdown of ILF3 promotes ferroptosis sensitivity in CRC cells

**Fig. 2:**
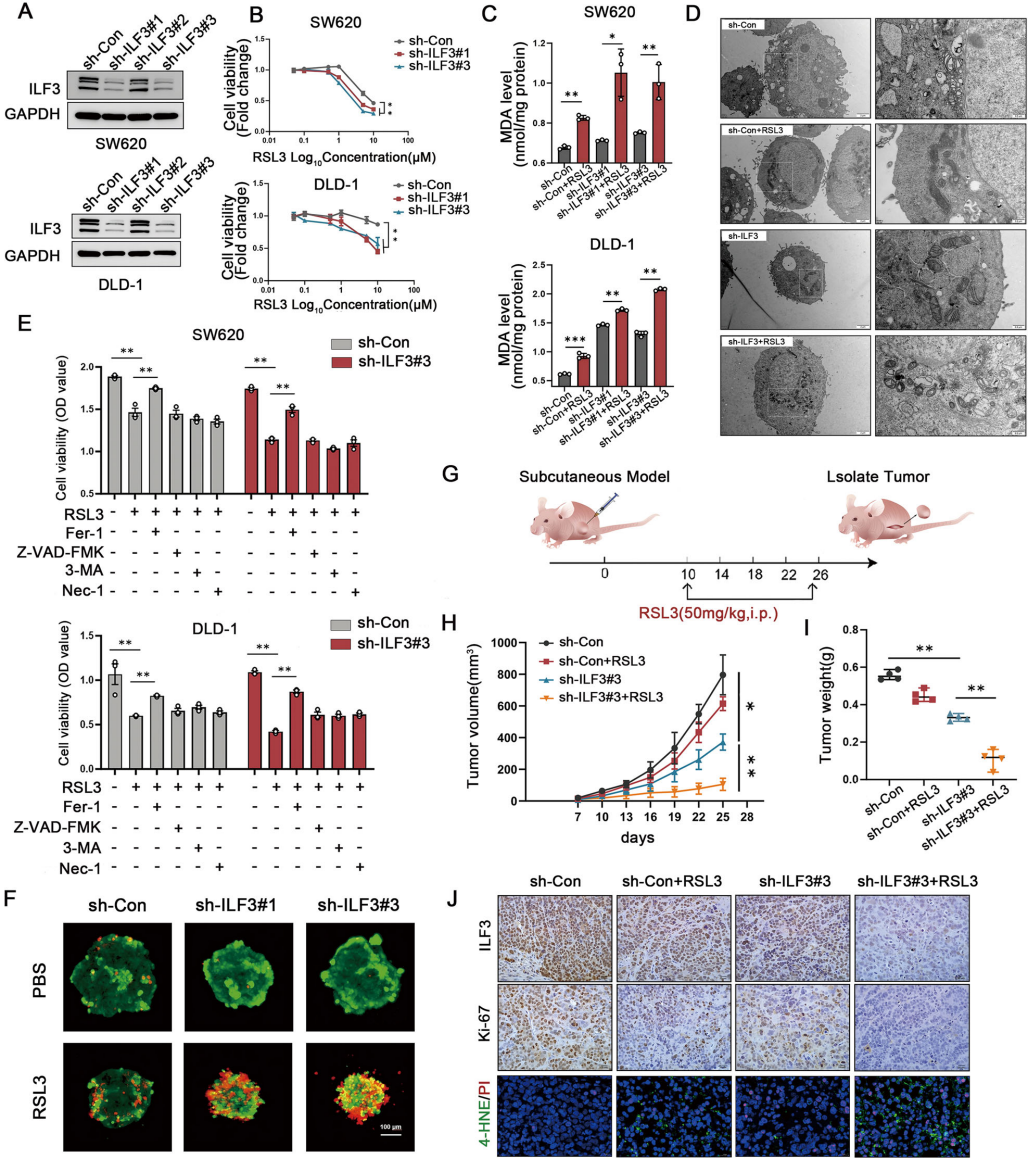
Knockdown of ILF3 promotes ferroptosis sensitivity in CRC cells. **A** Western blot analysis verifying the transfection efficiency of sh-ILF3 in SW620 and DLD-1 cells. **B** Cell viability of SW620 and DLD-1 cells in the sh-Con and sh-ILF3 groups following RSL3 treatment at different concentrations, assessed using a CCK-8 assay. **C** MDA levels in sh-Con and sh-ILF3 groups following RSL3 treatment (10 μM, 24 h). **D** Mitochondrial morphology in sh-Con and sh-ILF3 groups following RSL3 treatment (10 μM, 24 h). **E** Cell viability analysis of SW620 and DLD-1 cells treated with RSL3 (10 μM, 24 h) alone or in combination with ferrostatin-1 (1 μM, 24 h), Z-VAD-FMK (5 μM, 24 h), 3-methyladenine (2.5 mM, 24 h), or Necrostatin-1 (5 μM, 24 h) using a CCK-8 assay. **F** DLD-1 cells from the sh-ILF3 and sh-Con groups were cultured on agarose gel plates and treated with RSL3 (10 μM, 24 h) after seven days of culture. Cells were then stained using a Calcein/PI kit and imaged via confocal microscopy. Red represents PI staining, and green represents Calcein staining. **G** Schematic diagram illustrating the anticancer effect of RSL3 (50 mg/kg) combined with sh-ILF3#3 in a SW620 subcutaneous tumor model. **H** Tumor volume was measured every three days. **I** Tumor weight. J Immunohistochemical staining for ILF3 and Ki-67 and 4-HNE/PI co-staining. Scale bar = 20 μm.

To investigate the function of ILF3 in ferroptosis, we knocked down ILF3 expression in CRC cells (Fig. 2A). The CCK8 assay demonstrated that ILF3 loss significantly increased CRC cell sensitivity to RSL3-mediated cell death (Fig. 2B). Additionally, ILF3 knockdown significantly enhanced RSL3-induced MDA production and lipid peroxidation (Fig. 2C and Figure S2A). Transmission electron microscopy revealed that ILF3 knockdown cells exhibited enhanced mitochondrial shrinkage and increased membrane density after RSL3 treatment compared to sh-Con groups (Fig. 2D).

Importantly, ILF3 knockdown cells showed greater inhibition of proliferation following RSL3 treatment, an effect that was reversed by ferrostatin-1, a ferroptosis inhibitor. In contrast, other cell death inhibitors, including Z-VAD-FMK (apoptosis inhibitor), 3-methyladenine (autophagy inhibitor), and Necrostatin-1 (necrosis inhibitor), failed to reverse the effects, indicating that cell death caused by ILF3 knockdown was ferroptosis-dependent rather than mediated by other cell death pathways (Fig. 2E). Similar results were obtained in three-dimensional spheroid assays (Fig. 2F).

To further assess the impact of ILF3 depletion on ferroptosis in vivo, a subcutaneous xenograft mouse model was established. The inoculation and injection schematic is shown in Fig. 2G. As depicted in Fig. 2H and I, in the presence of RSL3, both tumor volume and weight were significantly reduced in the ILF3 knockdown group compared with the control group, while there was no significant difference in body weight of the mice between the groups (Fig. S2B). HE staining of mouse liver and kidney tissues indicated no apparent toxicity associated with RSL3 treatment (Fig. S2C). Additionally, Ki-67 and ILF3 expression levels were significantly decreased in the ILF3 knockdown groups treated with RSL3, and the population of 4-HNE and PI positive cells were increased (Fig. 2J). Collectively, these findings indicate that ILF3 knockdown promotes ferroptosis sensitivity in vivo.

### ILF3 is elevated in CRC and correlates with shorter survival

**Fig. 3:**
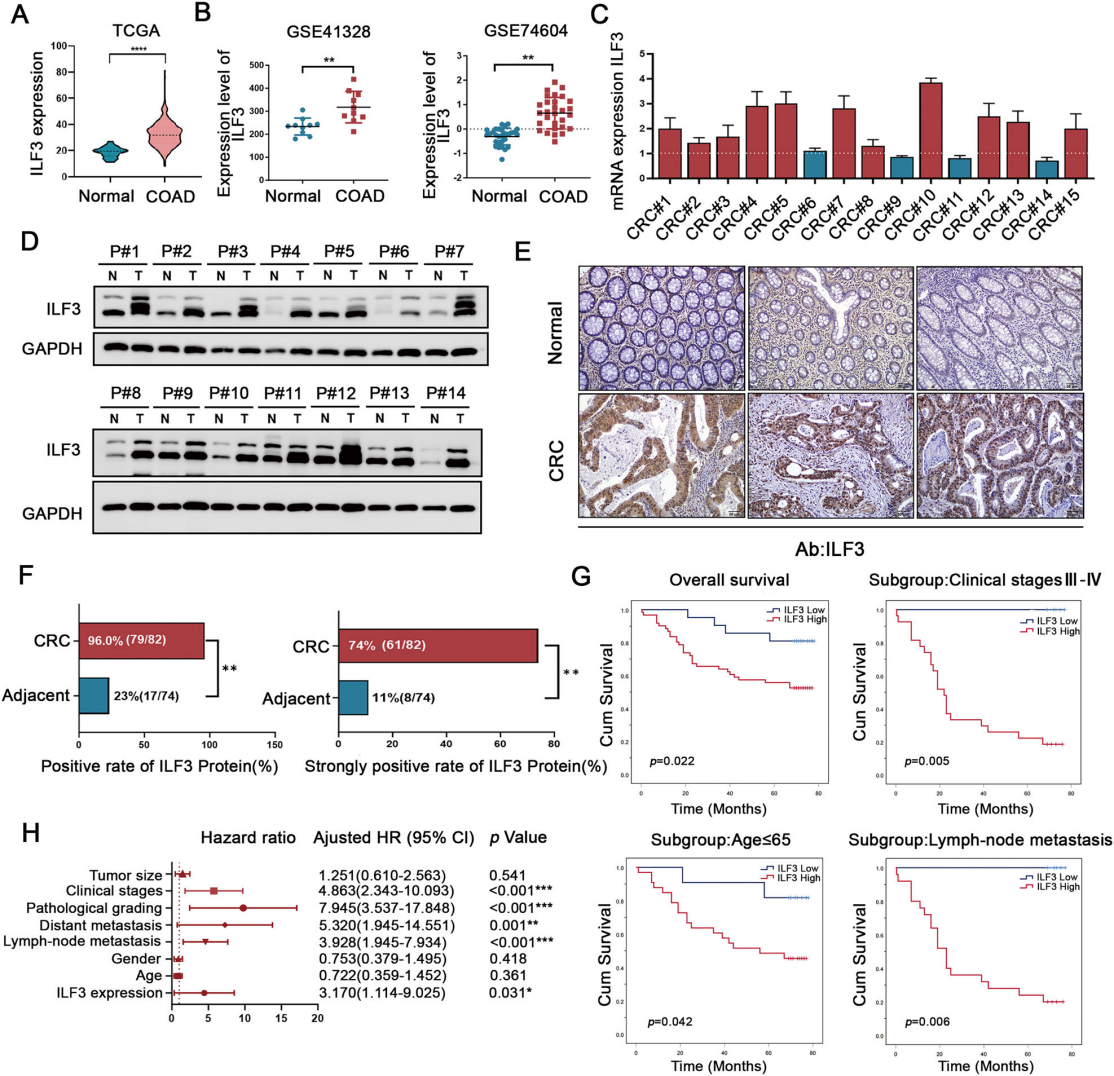
ILF3 is elevated in CRC and correlates with shorter survival. **A** ILF3 mRNA expression levels in colon adenocarcinoma tissue (*n* = 473) and normal tissue (*n* = 41) from the TCGA database. **B** ILF3 mRNA expression levels in colon adenocarcinoma and normal tissues from the GSE41328 and GSE74604 datasets. **C** RT-qPCR analysis of ILF3 mRNA expression levels in 15 paired colorectal cancer (CRC) and adjacent normal tissue samples. **D** Western blot analysis of ILF3 protein expression in 14 paired CRC and adjacent normal tissue samples. **E** Representative IHC images showing ILF3 expression in CRC and adjacent normal tissues. Scale bar = 50 μm. **F** Statistical analysis of ILF3 protein expression in CRC and adjacent normal tissues based on immunohistochemical staining results, *P* < 0.01. **G** Kaplan–Meier survival analysis showing the relationship between ILF3 expression levels and overall survival, with subgroup analysis based on clinical stage (III–IV), age (≤65 years), and lymph node metastasis. **H** Univariate Cox regression analysis of risk factors affecting the survival of patients with CRC.

Our study identified ILF3 as a crucial modulator of ferroptosis, suggesting its potential role in CRC progression through the regulation of ferroptosis evasion. To test this hypothesis, we assessed its clinical significance in patients with CRC. Analysis of the TCGA and GEO databases revealed that ILF3 mRNA levels were significantly upregulated in CRC tissues (Fig. 3A, B). RT-qPCR analysis of 15 paired CRC and adjacent nontumor tissues further confirmed that ILF3 mRNA expression was elevated in CRC tissues (Fig. 3C). To validate these findings at the protein level, we performed western blotting on 14 pairs of CRC tissue samples and found that ILF3 protein levels were higher in 13 pairs of the samples (Fig. 3D).

Additionally, immunohistochemical (IHC) staining of a tissue microarray (TMA) containing 82 CRC tissues and 74 adjacent non-tumor tissues revealed a higher ILF3 positivity rate (96.0%, 79/82; *P* < 0.01) and a higher strong positivity rate (74%, 61/82; *P* < 0.01) in CRC tissues. (Fig. 3E, F). ILF3 expression was significantly associated with tumor differentiation (*P* = 0.048) but not with lymph node metastasis or other clinicopathological factors (Supplementary Table 1). Kaplan–Meier survival analysis demonstrated that patients with CRC with high ILF3 expression had significantly shorter survival times than those with low ILF3 expression. This trend was also observed in subgroups characterized by clinical stage III or IV, age ≤ 65 years, and the presence of lymph node metastasis (Fig. 3G). Univariate Cox regression analysis identified lymph node metastasis (*P* < 0.001), distant metastasis (*P* = 0.001), pathological grade (*P* < 0.001), clinical stages (*P* < 0.001), and ILF3 expression (*P* = 0.031) as independent risk factors for patients with CRC (Fig. 3H). These findings indicate that ILF3 is upregulated in CRC and has predictive value for patient survival.

### ILF3 loss inhibits GSH synthesis by suppressing xCT-mediated cystine uptake

**Fig. 4:**
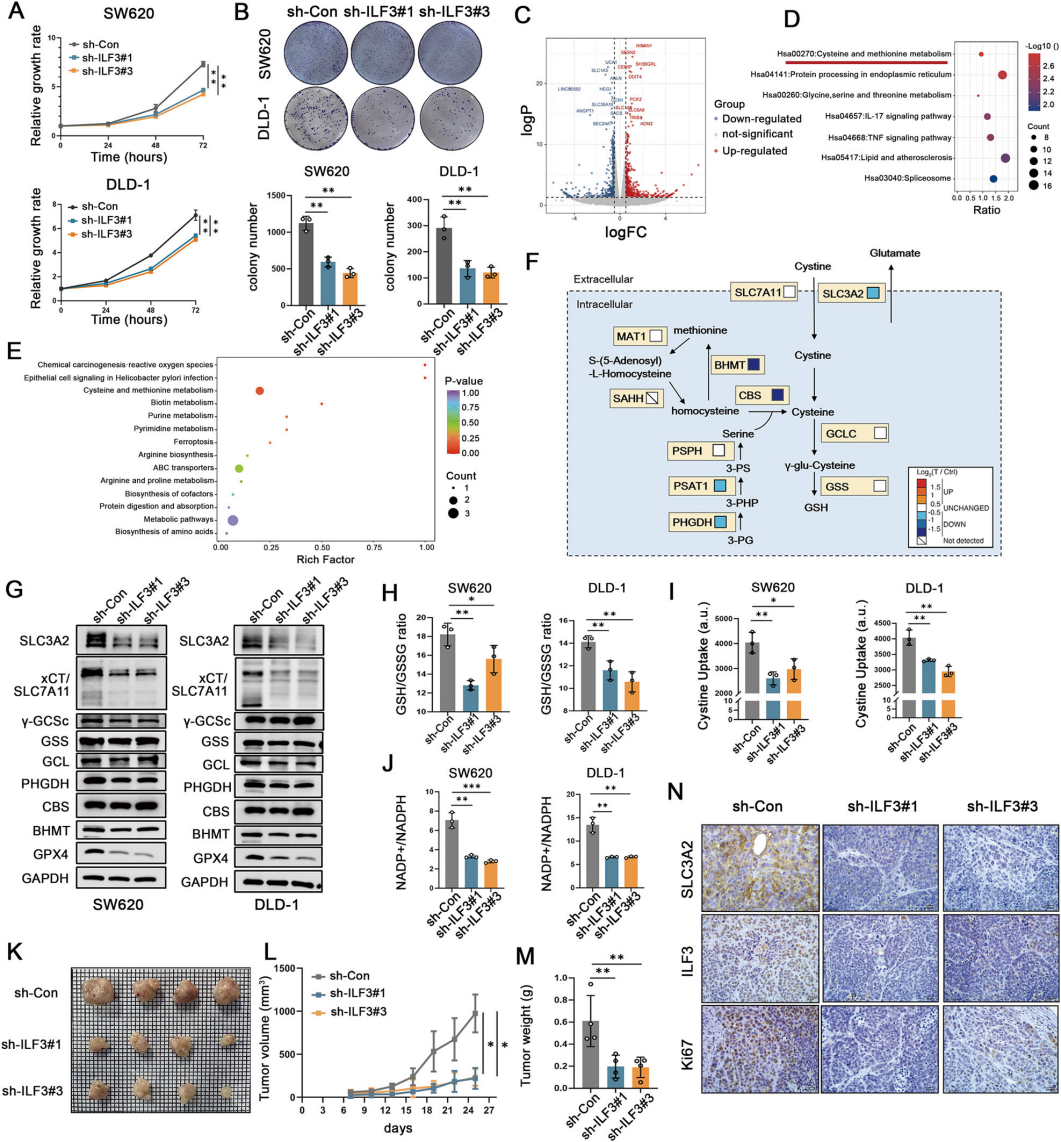
ILF3 loss inhibits GSH synthesis by suppressing xCT-mediated cystine uptake. **A**, **B** CCK-8 and colony formation assays assessing cell proliferation in ILF3 knockdown CRC cells. **C** Volcano plot presenting DEGs in ILF3 knockdown SW620 cells. **D** KEGG enrichment analysis of DEGs related to ILF3 knockdown. **E** Metabolic pathway enrichment analysis (MPWEA) of targeted amino acid metabolomics in ILF3 knockdown CRC cells. **F** Schematic representation of cysteine metabolism. The boxes to the right of each enzyme indicate changes in mRNA expression, with color coding corresponding to the level of log₂-fold change. **G** Western blot analysis of cysteine synthase (γ-GCSc, GSS, GCL, BHMT, CBS, and PHGDH) and cystine transporters (SLC7A11 and SLC3A2) in ILF3 knockdown CRC cells. **H–J** Intracellular GSH levels, cystine uptake ability, and NADPH levels in ILF3 knockdown cells. **K** Representative tumor images from SW620 cell-transplanted nude mice. **L** Tumor volume measured every 3 days. **M** Tumor weight. **N** Immunohistochemical staining of ILF3, SLC3A2 and Ki-67. Scale bar = 20 μm.

To investigate the molecular mechanisms through which ILF3 regulates ferroptosis, we first assessed the effects of ILF3 depletion on CRC cell proliferation. ILF3 knockdown significantly reduced cell viability, colony formation, and DNA synthesis. (Figs. 4A, B and S3A). Next, we performed RNA-seq in ILF3 knockdown cells, and identified 1, 058 DEGs (Fig. 4C). Gene set enrichment analysis revealed that cysteine and methionine metabolism was one of the most relevant pathways (Fig. 4D). Abnormal amino acid metabolism is a key aspect of tumor metabolic reprogramming and a crucial factor in ferroptosis [19]. To further confirm whether ILF3 could regulate amino acid metabolism, particularly cysteine and methionine metabolism, we performed targeted amino acid metabolomics. Our metabolomic analysis revealed that ILF3 knockdown led to decreased levels of L-cysteine, N8-acetylspermidine, urea, and S-(5′-adenosyl)-L-homocysteine, while increasing the levels of ethanolamine and TRP-GLU (Fig. S3B). In addition, Metabolic pathway enrichment analysis indicated strong enrichment in cysteine and methionine metabolism, as well as ferroptosis-related pathways (Fig. 4E). A combined transcriptomic and metabolomic analysis further identified broad deregulation of cysteine and methionine metabolism through the regulation of SLC3A2, BHMT, CBS, PSAT1, and PHGDH (Fig. 4F). Western blot analysis confirmed that ILF3 knockdown downregulated the expression of SLC7A11, SLC3A2, and GPX4, whereas BHMT, CBS, PHGDH, GCL, GSS, and γ-GCSc showed no significant changes (Fig. 4G). Given that SLC7A11 and SLC3A2 are subunits of the xCT cystine/glutamate transport system, which facilitates cystine uptake in cells [20], we hypothesized that ILF3 knockdown inhibits intracellular GSH synthesis by suppressing xCT transporter-mediated cystine uptake.

Consistent with our hypothesis, ILF3 knockdown decreased cystine uptake, GSH synthesis, and the NADP + /NADPH ratio in CRC cells (Fig. 4H–J), while other ferroptosis-related factors, including ACSL4, FTH1, and TFRC, remained unaffected (Fig. S3C). A nude mouse xenograft model further confirmed that ILF3 knockdown significantly inhibited tumor volume and weight (Fig. 4K–M). IHC staining revealed that ILF3 knockdown led to a decrease in SLC3A2 and Ki-67 expression (Fig. 4N). Collectively, these findings demonstrate that ILF3 loss inhibits GSH synthesis by suppressing xCT-mediated cystine uptake.

### ILF3 modulates SLC3A2 mRNA stability by directly binding to the 3’UTR

**Fig. 5:**
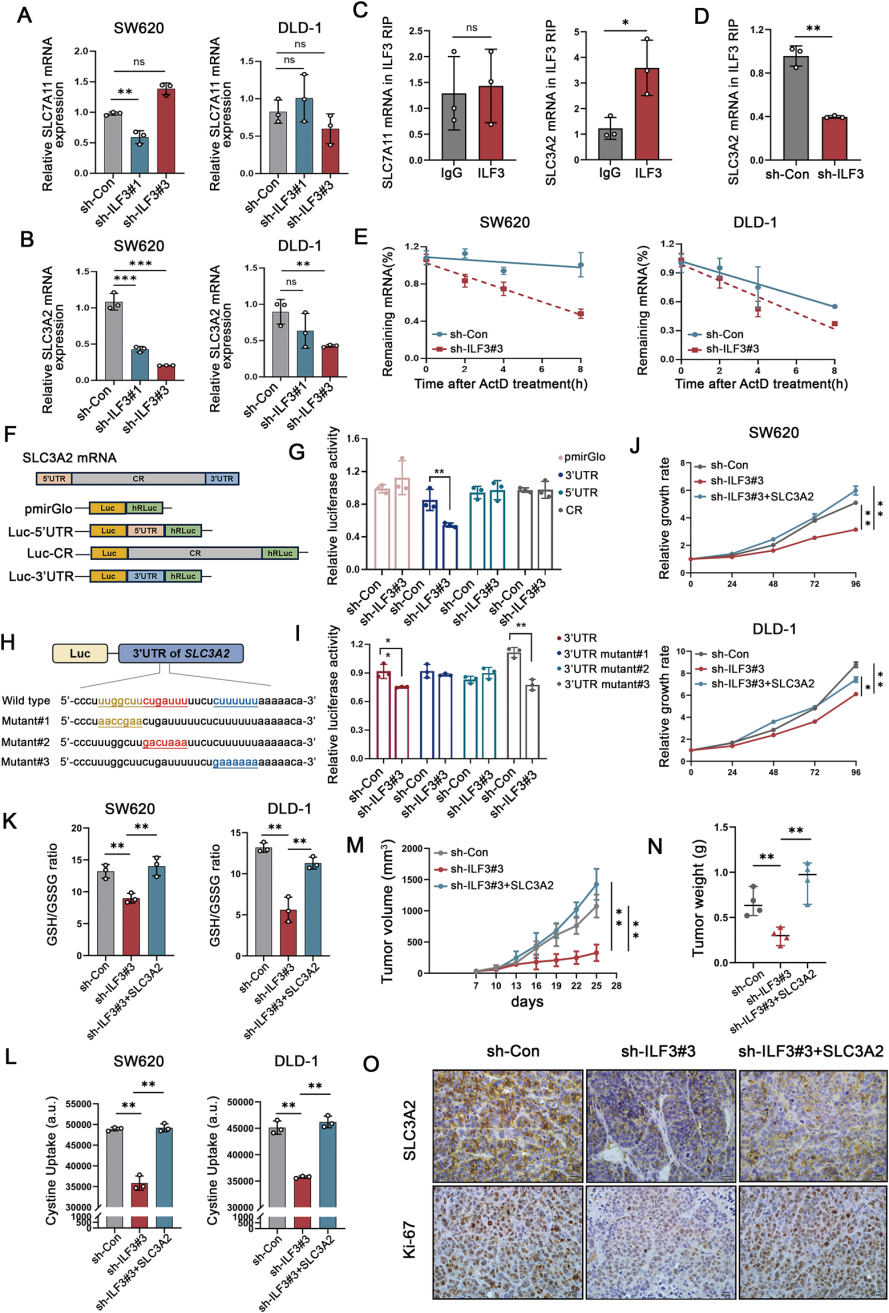
ILF3 modulates SLC3A2 mRNA stability by directly binding to the 3’ UTR. **A**, **B** RT-qPCR analysis of SLC7A11 and SLC3A2 mRNA levels in ILF3 knockdown SW620 and DLD-1 cells. **C** RIP assay evaluating the relative enrichment of SLC7A11 and SLC3A2 mRNA in ILF3-RNA binding complexes; anti-IgG served as a negative control. **D** RIP assay evaluating the relative enrichment of SLC3A2 mRNA in ILF3 knockdown cells. **E** mRNA stability analysis of SLC3A2 in ILF3 knockdown cells after ActD (5 μg/mL) treatment. **F**, **G** Schematic representation of the SLC3A2 luciferase reporter plasmid and relative luciferase activities in ILF3 knockdown cells, calculated as the ratio of firefly to Renilla luciferase activity. **H**, **I** Schematic representation of wild-type and three mutant SLC3A2 3’ UTR luciferase reporter plasmids and relative luciferase activities in ILF3 knockdown cells. **J** Cell viability in ILF3 knockdown cells with or without SLC3A2 overexpression. **K**, **L** GSH levels and cystine uptake in ILF3 knockdown cells with or without SLC3A2 overexpression. **M** Xenograft mouse model constructed using ILF3 knockdown cells with or without SLC3A2 overexpression. Tumor volume was measured every 3 days. **N** Tumor weight. **O** Immunohistochemical staining of SLC3A2 and Ki-67. Scale bar = 20 μm.

To further investigate the molecular mechanisms by which ILF3 regulates xCT, we examined whether ILF3 modulates SLC3A2 and SLC7A11 expression. RT-qPCR results showed that ILF3 knockdown significantly reduced the mRNA levels of SLC3A2, but not SLC7A11 (Fig. 5A, B). Given that ILF3 is an RBP known to regulate mRNA stability [15, 21, 22], we examined whether ILF3 directly binds to SLC7A11 or SLC3A2 mRNA. RIP assays demonstrated that SLC3A2 mRNA was significantly enriched in ILF3 immunoprecipitates, whereas SLC7A11 was not (Fig. 5C). ILF3 knockdown markedly reduced its binding to SLC3A2 mRNA (Fig. 5D).

An actinomycin D assay further demonstrated that ILF3 loss reduced the half-life of SLC3A2 mRNA (Fig. 5E). To identify the specific binding site, we generated three luciferase reporter constructs containing different fragments of SLC3A2 mRNA: coding region, 5′ UTR, and 3′ UTR (Fig. 5F). Luciferase assays revealed that ILF3 knockdown significantly reduced luciferase activity in the SLC3A2-3′ UTR construct, whereas the SLC3A2-5′ UTR and SLC3A2-CR constructs showed no significant changes, indicating that ILF3 binds to the 3′ UTR of SLC3A2 mRNA (Fig. 5G). To further confirm the binding specificity, we used the RBPsuite database to predict three putative ILF3 binding sites within the SLC3A2 3′ UTR. We then constructed three mutant luciferase plasmids (3′ UTR mutant#1, 3′ UTR mutant#2, 3′ UTR mutant#3) (Fig. 5H) and tested their response to ILF3 knockdown. ILF3 knockdown failed to inhibit luciferase activity in the 3′ UTR mutant#1 and 3′ UTR mutant#2 constructs, confirming that these regions are the binding sites for ILF3 (Fig. 5I). These findings demonstrate that ILF3 stabilizes SLC3A2 mRNA by binding to its 3′ UTR.

To further validate whether ILF3 modulates cystine uptake and GSH synthesis via SLC3A2, we overexpressed SLC3A2 in ILF3 knockdown cells (Fig. S4A) and examined its effects on cell proliferation, cystine uptake, and GSH synthesis. SLC3A2 overexpression successfully reversed the inhibitory effects of ILF3 knockdown on cell proliferation (Figs. 5J and S4B). Additionally, GSH synthesis and cystine uptake were restored by SLC3A2 overexpression (Fig. 5K, L). BODIPY C11 staining showed that overexpression of SLC3A2 reduced ILF3 knockdown-induced lipid peroxidation (Fig. S4C). These results were further confirmed in xenograft models, where SLC3A2 overexpression significantly increased tumor volume, tumor weight, and Ki-67 expression in ILF3 knockdown tumors (Fig. 5M–O). Collectively, these findings indicate that ILF3 knockdown inhibits cell proliferation, cystine uptake, and GSH synthesis by downregulating SLC3A2 expression.

### ILF3 and SLC3A2 are co-expressed in CRC tissues

**Fig. 6:**
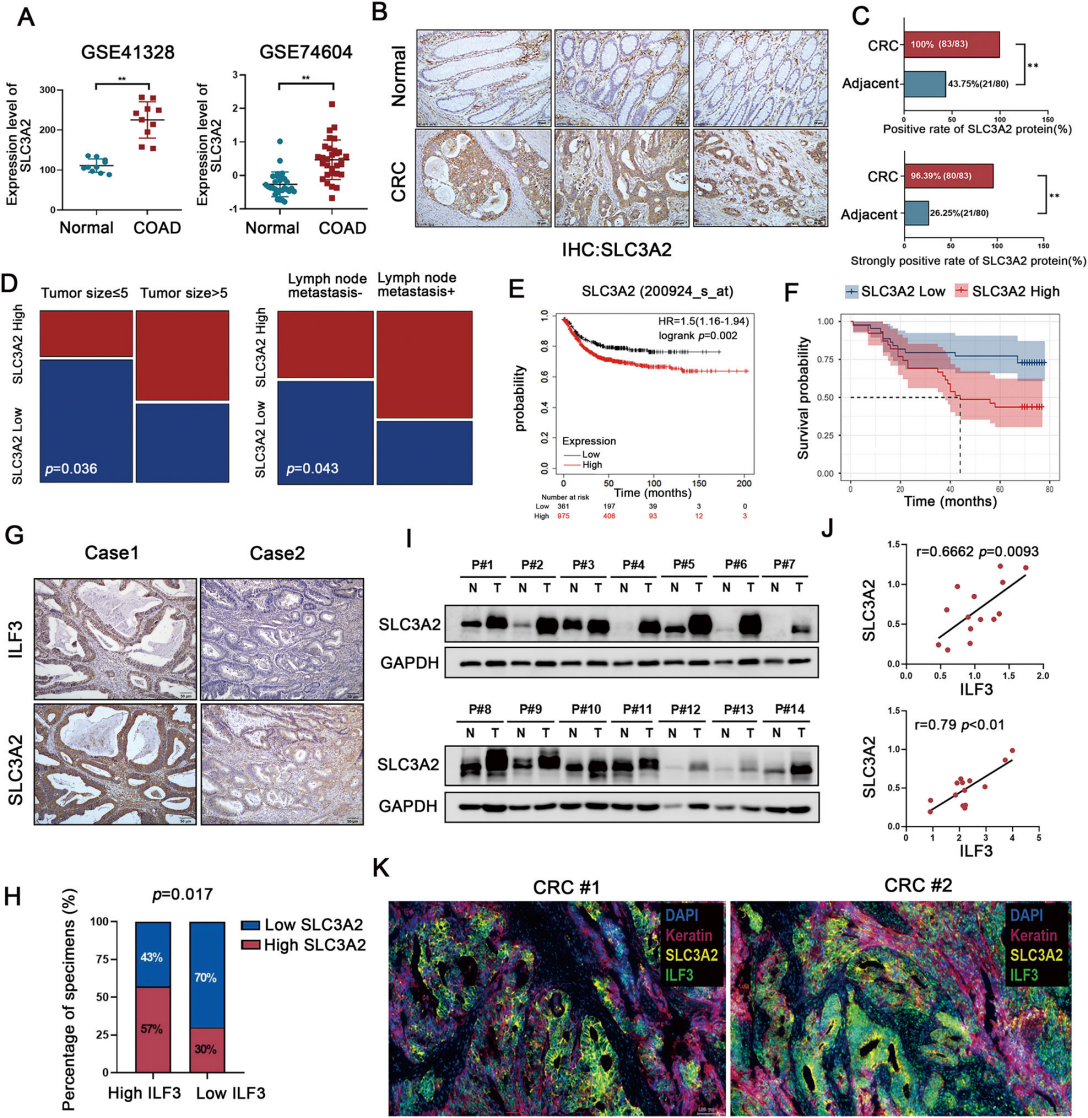
ILF3 and SLC3A2 are co-expressed in CRC tissues. **A** The GSE41328 and GSE74604 datasets summarize the expression levels of SLC3A2 mRNA in colon adenocarcinoma and normal tissues. **B** Representative IHC images showing SLC3A2 expression in colorectal cancer (CRC) and adjacent normal tissues. Scale bar = 20 μm. **C** Statistical analysis of the positive and strongly positive rates of SLC3A2 protein expression in CRC and adjacent normal tissues based on immunohistochemical (IHC) staining results, *P* < 0.01. **D** Correlation analysis of SLC3A2 protein expression levels with tumor size (*P* = 0.036) and lymph node metastasis (*P* = 0.043). **E** Kaplan–Meier survival curve showing overall survival based on SLC3A2 expression levels obtained from KM-plotter database (Affymetrix ID = 200924_s_at). **F** Overall survival curves for SLC3A2 protein expression profiles. **G**, **H** IHC staining experiments showing that ILF3 and SLC3A2 staining areas co-localized (**G**), and their IHC staining scores were positively correlated (**H**). **I** Western blot analysis of SLC3A2 protein expression in 14 paired CRC and adjacent normal tissue samples. **J** Correlation analysis of ILF3 and SLC3A2 protein expression levels detected by western blot, quantified using Image J. **K** Representative multiplex immunostaining image of a CRC tissue sample (keratin, red; ILF3, green; SLC3A2, yellow; DAPI, blue). Keratin was used to label tumor cells, and DAPI was used to highlight nuclei.

To further investigate the relationship between ILF3 and SLC3A2 in CRC, we first analyzed SLC3A2 expression in CRC tissues. Analysis of the GEO database revealed upregulated expression of SLC3A2 mRNA in CRC tissues (Fig. 6A). IHC staining showed that SLC3A2 exhibited significantly elevated positive (100%, 83/83) and strongly positive (96.39%, 80/83) expression rates in CRC tissues compared to adjacent normal tissues. (*P* < 0.01) (Fig. 6B, C). Further analysis of clinical relevance revealed that high SLC3A2 expression was positively correlated with tumor size and lymph node metastasis (Fig. 6D). Additionally, patients with high SLC3A2 mRNA and protein levels exhibited shorter survival times (Fig. 6E, F).

Subsequently, we correlated the expression of ILF3 and SLC3A2 at the transcriptional and translational levels. IHC and western blotting analyses showed a significant positive correlation between ILF3 and SLC3A2 protein expression (Fig. 6G–J). Consistently, a consistent positive correlation was also observed between SLC3A2 mRNA levels and ILF3 protein abundance (*P* < 0.05) (Fig. S5A, B). Furthermore, multiplex immunofluorescence staining demonstrated colocalization of ILF3 and SLC3A2 in CRC tissue samples (Fig. 6K). Together, these findings indicate that ILF3 and SLC3A2 expression levels are positively correlated in CRC tissues.

### TNF-α accelerates cystine uptake and GSH synthesis by upregulating ILF3 expression

**Fig. 7:**
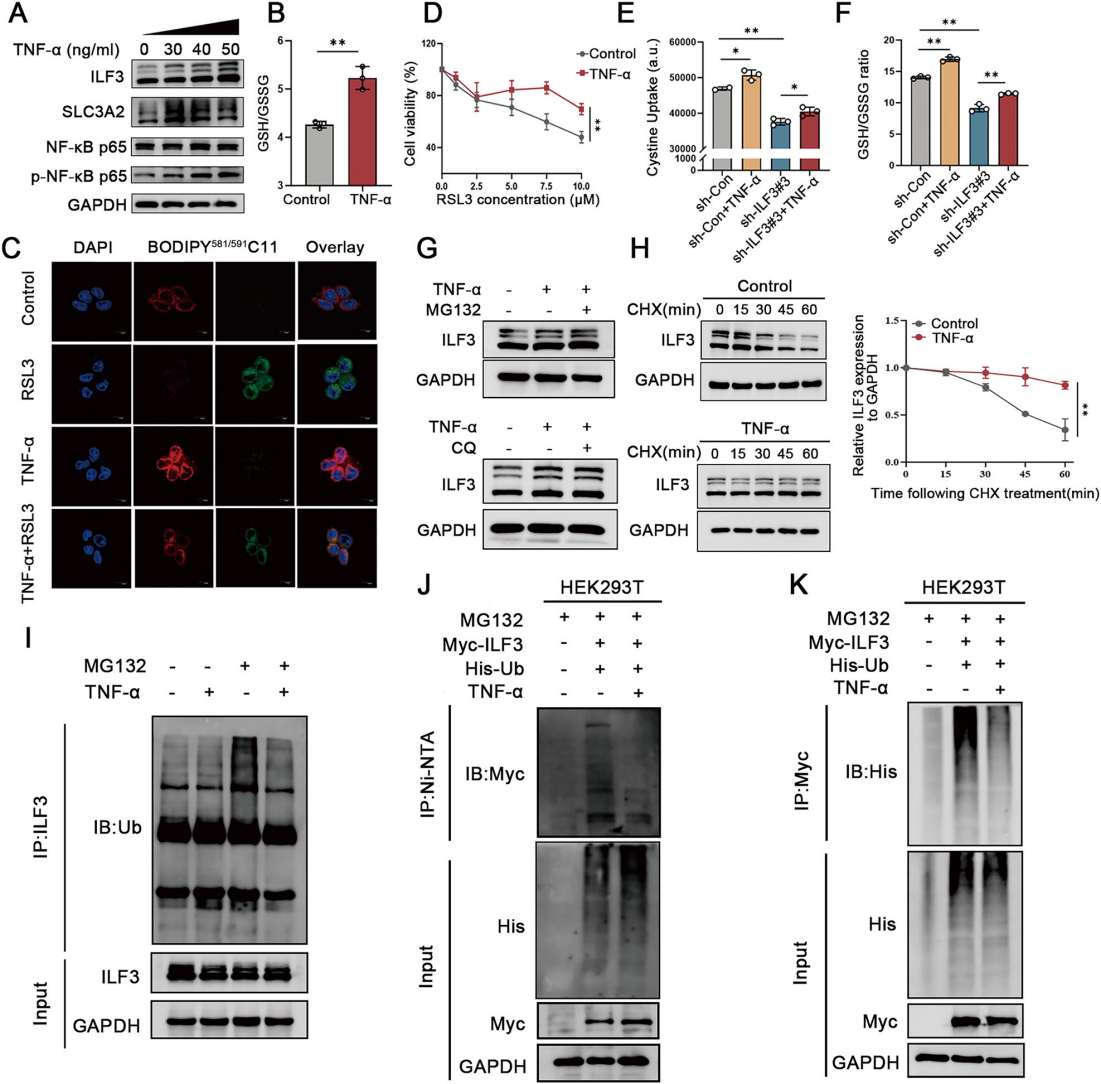
TNF-α accelerates cystine uptake and GSH synthesis by upregulating ILF3 expression. **A** SW620 cells were treated with different concentrations of TNF-α for 24 h, and protein expression levels of ILF3, SLC3A2, NF-κB p65, and p-NF-κB p65 were analyzed by western blot. **B** SW620 cells were treated with TNF-α (40 ng/mL) for 24 h, and the GSH/GSSG ratio was determined using a GSH and GSSG assay kit. **C** SW620 cells were treated with TNF-α (40 ng/mL) and RSL3 (10 μM) alone or in combination for 24 h, and lipid peroxidation levels were detected using a BODIPY (581/591) C11 probe. Scale bar = 10 μm. **D** SW620 cells were pretreated with TNF-α (40 ng/mL) for 12 h, followed by RSL3 treatment at different concentrations for 24 h. Cell viability was assessed using a CCK-8 assay. **E**, **F** Cystine uptake and GSH levels were measured in sh-Con and sh-ILF3#3 SW620 cells with or without TNF-α (40 ng/mL, 24 h). **G** SW620 cells were pretreated with TNF-α (40 ng/mL) for 24 h, then incubated with MG132 (20 μM, 4 h) or chloroquine (10 μM, 24 h), and ILF3 protein expression levels were analyzed by western blot. **H** SW620 cells were treated with TNF-α (40 ng/mL) for 24 h and co-treated with cycloheximide (60 μg/mL) for different durations before harvesting. ILF3 protein expression levels were analyzed by Western blot. **I** SW620 cells were treated with TNF-α (40 ng/mL) for 24 h, incubated with MG132 (20 μM) for 4 h before harvesting, and then subjected to in vivo ubiquitination analysis. **J** HEK293T cells were co-transfected with Myc-ILF3 and His-Ub plasmids for 48 h, treated with TNF-α (40 ng/mL, 24 h) and MG132 (20 μM, 4 h), and the protein mixture was purified using Ni-NTA magnetic beads and analyzed by western blot using an anti-Myc antibody. **K** HEK293T cells were co-transfected with Myc-ILF3 and His-Ub for 48 h, treated with TNF-α (40 ng/mL, 24 h) and MG132 (20 μM, 4 h), and subjected to in vivo ubiquitination analysis using an anti-His antibody.

TNF-α exerts tumor-protective functions by activating pro-survival signaling pathways (e.g., NF-κB), inducing a tumor-associated inflammatory microenvironment, suppressing tumor immune responses, and promoting tumor cell survival, invasion, and drug resistance [23]. Our previous analysis revealed that ILF3-related DEGs were enriched in the TNF signaling pathway (Fig. 4D). Accordingly, we hypothesized that TNF-α stimulation contributes to elevated ILF3 expression in CRC tissues.To test this hypothesis, SW620 cells were treated with different concentrations of TNF-α, and we observed up-regulated of ILF3 and SLC3A2 expression over a range of concentrations and time. (Figs. 7A and S6A). Similar results were obtained in HCT-116 cells (Fig. S6B). However, treatment with another inflammatory cytokine, IL-6, did not significantly upregulate ILF3 protein expression (Fig. S6C). Next, we investigated whether TNF-α modulates ferroptosis in colorectal cancer cells. The results showed that TNF-α treatment enhanced cell viability and increased GSH levels while reducing the sensitivity of CRC cells to RSL3-induced ferroptosis (Fig. 7B–D and Fig. S6D). Notably, ILF3 knockdown partially abolished the effects of TNF-α on GSH synthesis and cystine uptake (Fig. 7E, F), indicating that TNF-α promotes these processes via ILF3 upregulation.

To elucidate the mechanisms by which TNF-α enhances ILF3 expression, we assessed ILF3 mRNA levels following TNF-α treatment. Interestingly, TNF-α did not alter ILF3 mRNA expression (Fig. S6E), suggesting that its regulation occurs primarily at the post-transcriptional level. Notably, treatment with the proteasome inhibitor MG132 increased ILF3 expression in TNF-α-treated cells, whereas treatment with the lysosomal inhibitor chloroquine had no effect (Fig. 7G). In addition, cycloheximide assays showed that TNF-α treatment prolonged ILF3 protein half-life (Fig. 7H), indicating that TNF-α regulates ILF3 expression via a proteasome-dependent mechanism. We then performed a ubiquitination assay to assess the interaction between ILF3 and ubiquitin under TNF-α stimulation. Our findings demonstrated that TNF-α treatment significantly reduced endogenous ILF3 ubiquitination (Fig. 7I). Additionally, Ni-NTA pull-down assays under denaturing conditions in HEK293T cells co-transfected with Myc-ILF3 and His-Ub further confirmed that TNF-α stimulation decelerated ILF3 ubiquitination (Fig. 7J). Western blotting using an anti-Myc antibody further demonstrated that TNF-α reduced polyubiquitination of ILF3 (Fig. 7K).

### TNF-α upregulates ILF3 expression by inhibiting TRIM17-mediated ubiquitination

**Fig. 8:**
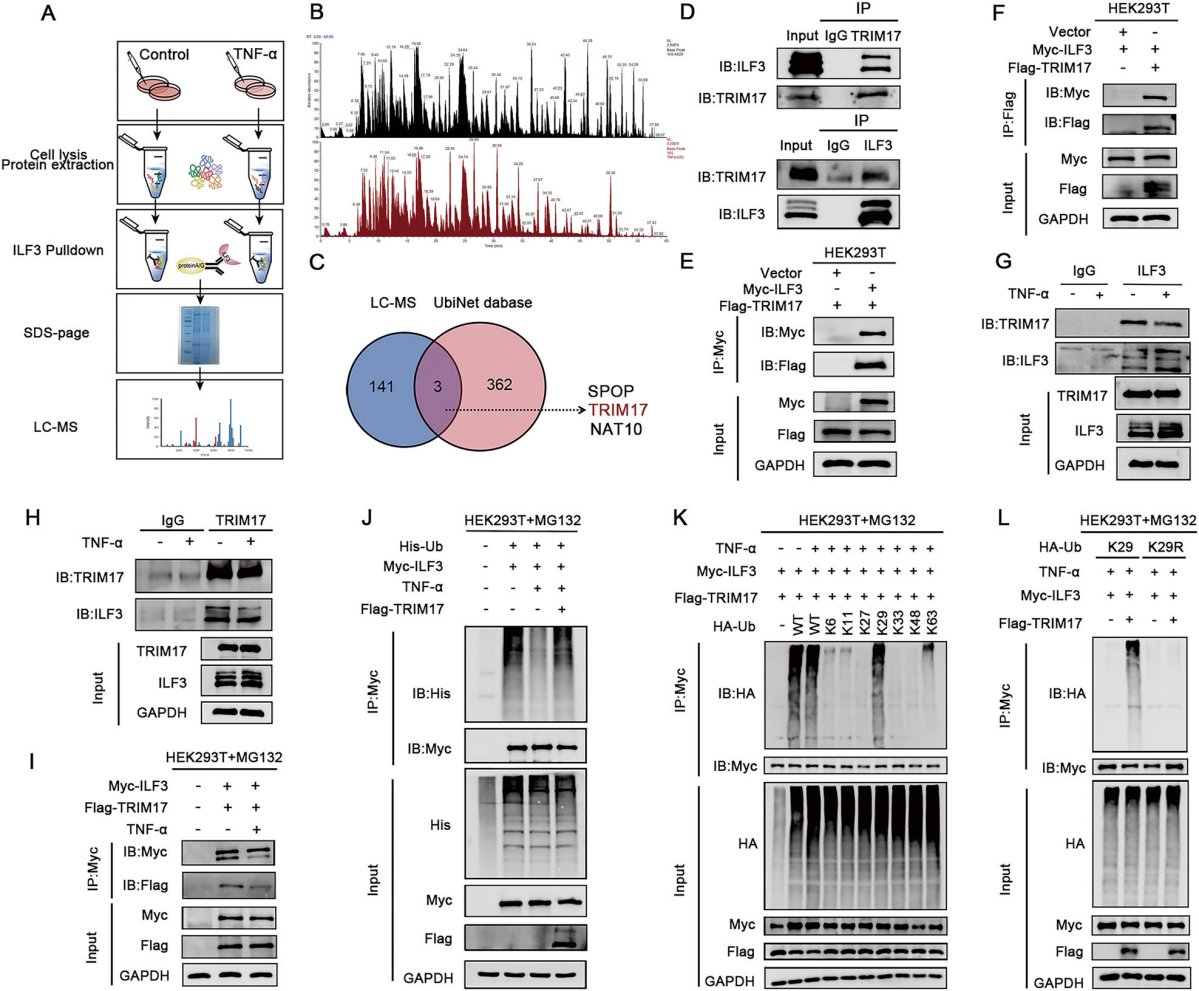
TNF-α upregulates ILF3 expression by inhibiting TRIM17-mediated ubiquitination. **A** Workflow for differential protein identification via pull-down of ILF3 followed by LC/MS analysis. **B** Protein mass spectrometry identification results. **C** Venn diagram showing the intersection of 141 differentially expressed proteins identified in SW620 cells treated with TNF-α via LC/MS and 362 E3 ubiquitin ligases retrieved from the UbiNet database. **D** Co-IP analysis of SW620 cell lysates using an anti-ILF3 antibody or an anti-TRIM17 antibody and control IgG to detect interactions between ILF3 and TRIM17 proteins. **E**, **F** HEK293T cells were co-transfected with Flag-TRIM17 or a control vector and Myc-ILF3 for 48 h, followed by IP and IB analyses. **G**, **H** SW620 cells treated with TNF-α (40 ng/mL, 24 h) were harvested, and Co-IP was performed using an anti-ILF3 or anti-TRIM17 antibody and control IgG to determine whether TNF-α affects the interaction between ILF3 and TRIM17 proteins. **I** HEK293T cells were co-transfected with Flag-TRIM17 and Myc-ILF3 for 48 h, treated with TNF-α (40 ng/mL, 24 h) and MG132 (20 μM, 4 h), and subjected to IP and IB analysis. **J** HEK293T cells were co-transfected with Flag-TRIM17, His-Ub, and Myc-ILF3, treated with TNF-α (40 ng/mL, 24 h) and MG132 (20 μM, 4 h), and subjected to IP and IB analysis to determine whether TNF-α affects ILF3 ubiquitination by TRIM17. **K** HEK293T cells were transfected with Myc-ILF3 and Flag-TRIM17 together with ubiquitin WT or different ubiquitin mutants. After treatment with TNF-α (40 ng/mL, 24 h) and MG132 (20 μM, 4 h), the cells were collected and subjected to western blot assays. **L** HEK293T cells were transfected with Myc-ILF3 and Flag-TRIM17 together with ubiquitin K29R mutant. After treatment with TNF-α (40 ng/mL, 24 h) and MG132 (20 μM, 4 h), the cells were collected and subjected to western blot assays.

E3 ubiquitin ligases are crucial mediators of ubiquitination, facilitating the transfer of ubiquitin to lysine residues on target substrates [20]. To identify the specific E3 ubiquitin ligase involved in ILF3 ubiquitination, ILF3 was pulled down from cells with or without TNF-α treatment, followed by liquid chromatography-mass spectrometry analysis (Fig. 8A, B). Three candidate E3 ubiquitin ligases were identified: SPOP, TRIM17, and NAT10 (Fig. 8C). We then evaluated the interaction between ILF3 and TRIM17. Endogenous binding between ILF3 and TRIM17 was detected by Co-immunoprecipitation (Co-IP) assay (Fig. 8D). Additionally, exogenous co-immunoprecipitation in HEK293T cells confirmed that Flag-tagged TRIM17 and Myc-tagged ILF3 formed complexes (Fig. 8E, F). Further immunoprecipitation assays demonstrated that TNF-α treatment significantly reduced both endogenous and exogenous binding between TRIM17 and ILF3 (Fig. 8G–I).

Importantly, TRIM17 overexpression enhanced ILF3 ubiquitination even in TNF-α-treated cells (Fig. 8J). Taken together, these results establish that TNF-α disrupts the binding between ILF3 and TRIM17, leading to a decrease in ILF3 ubiquitination. To determine the specific ubiquitin chains involved in ILF3 ubiquitination, We co-transfected Myc-ILF3 and Flag-TRIM17 together with ubiquitin-WT or linkage-specific ubiquitin mutants. The results showed that TRIM17-mediated K29-linked polyubiquitination of ILF3 (Fig. 8K). Additionally, the ubiquitin mutant K29R, in which K29 was replaced by arginine (R), was unable to support this modification of ILF3 (Fig. 8L).

## Discussion

Gene expression regulation is a complex process that coordinates cellular responses through various mechanisms, including transcriptional, post-transcriptional, and post-translational modifications. Among these, post-transcriptional regulation plays a particularly significant role as it affects RNA stability, structure, and function [24]. RBPs are key regulators in this framework, modulating gene expression at the post-transcriptional level [25–27]. Abnormal RBP expression has been linked to tumor initiation and progression, underscoring their potential as targets for novel therapeutic strategies [28].

The central findings of our study identify ILF3 as a key RBP that regulates both the abundance and stability of SLC3A2 mRNA, thereby influencing the sensitivity of CRC cells to ferroptosis. Although the mechanisms underlying ferroptosis have received increasing attention, research on the role of RBPs in modulating this process remains limited. Previous studies demonstrated that the RBP RBMS1 binds to SLC7A11 mRNA to enhance its translation, contributing to ferroptosis resistance in lung cancer [29]. Similarly, TTP destabilizes ATG16L1 mRNA, thereby promoting ferroptosis resistance [30]. Our findings suggest that ILF3 serves as a crucial modulator of ferroptosis by enhancing the stability of SLC3A2 mRNA via binding to its 3’ UTR. This is particularly significant as it highlights ILF3 not only as a stabilizing factor but also as a key regulator of metabolic pathways associated with cancer progression.

Recent studies have emphasized the essential role of amino acid metabolism in regulating ferroptosis [31]. Our findings support the notion that ILF3 is involved in amino acid metabolism, particularly in the regulation of serine and cysteine synthesis [15, 32]. Notably, our transcriptome sequencing and metabolomic analyses revealed that ILF3 plays a role in cysteine and methionine metabolism, further establishing its importance in modulating oxidative stress through GSH synthesis. We confirmed that ILF3 knockdown significantly decreases cystine uptake, primarily by downregulating SLC3A2 and SLC7A11 expression. This regulatory mechanism illustrates how ILF3 not only promotes mRNA stability but also primes cellular responses to protect against ferroptosis by enhancing GSH synthesis.

In CRC, ILF3 expression is elevated and correlated with poor patient prognosis. A significant finding of our study is the link between ILF3 upregulation and TNF-α stimulation. Our results indicate that TNF-α enhances cystine uptake and GSH synthesis, ultimately promoting resistance to ferroptosis. This is consistent with previous observations that TNF-α/TNFR2 signaling enhances cystine uptake to protect cells from ferroptosis [33]. Furthermore, we identified ILF3’s role in TNF-α-mediated inhibition of ferroptosis. The interaction between ILF3 and TRIM17 suggests a complex regulatory network in which TRIM17 ubiquitinates ILF3, a process that is inhibited by TNF-α treatment. While previous reports suggested that ILF3 may be phosphorylated via the ERK pathway to inhibit SPOP-mediated ubiquitination [15], our study did not confirm ILF3 phosphorylation under TNF-α stimulation. This discrepancy highlights the need for further investigation into the molecular mechanisms involved.

In conclusion, the interaction between ILF3 and SLC3A2 mRNA functions as a critical event in regulating cystine uptake and GSH synthesis, which in turn modulates ferroptosis resistance in CRC cells. TNF-α modulates ferroptosis resistance in CRC cells by upregulating ILF3 through the inhibition of TRIM17-mediated ubiquitination. Our findings indicate that ILF3 may serve as a potential therapeutic target for ferroptosis-focused cancer treatment.

## Supplementary information

Supplementary Figures

Supplementary Methods

Supplementary tables

Uncropped original western blots

## Data Availability

The data that support the findings of this study are available from the corresponding author upon reasonable request.
