# Supplementary material for: ILF3 promotes colorectal cancer cell resistance to ferroptosis by enhancing cysteine uptake and GSH synthesis via stabilizing SLC3A2 mRNA
Source: Cell Death Dis. 2025 Jul 23;16(1):549. doi: 10.1038/s41419-025-07872-x (PMC12284142; doi:10.1038/s41419-025-07872-x)
Supplement: Supplementary file 1 — Supplementary Figures [file 41419_2025_7872_MOESM1_ESM.docx]

**Fig. S1, related to Fig 1**


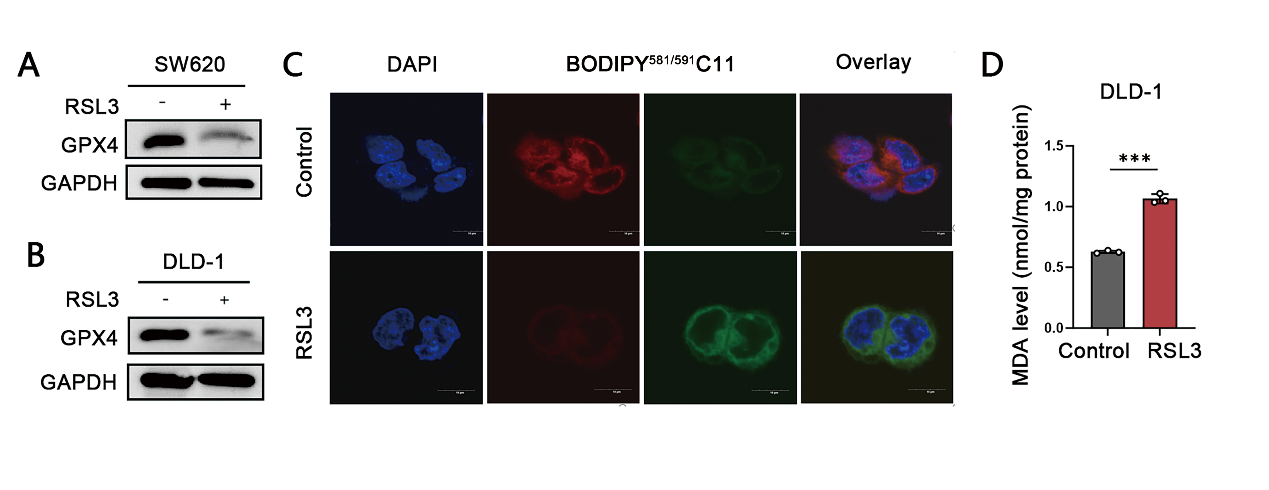


**Fig. S1. ILF3 is decreased in RSL3-mediated ferroptosis. A, B** The protein level of GPX4 in RSL3-induced (10μM,24h) SW620 and DLD-1 cells was detected by western blot assay. **C** Lipid peroxidation levels in DLD-1 cells treated with RSL3 (10 μM, 24 h) were detected using a BODIPY (581/591) C11 probe. Scale bar = 10 μm. Red represents reductive status, green represents oxidative status, and blue represents DAPI staining. **D** MDA levels in DLD-1 cells treated with RSL3 (10 μM, 24 h) compared to the control group.

**Fig S2, related to Fig 2**


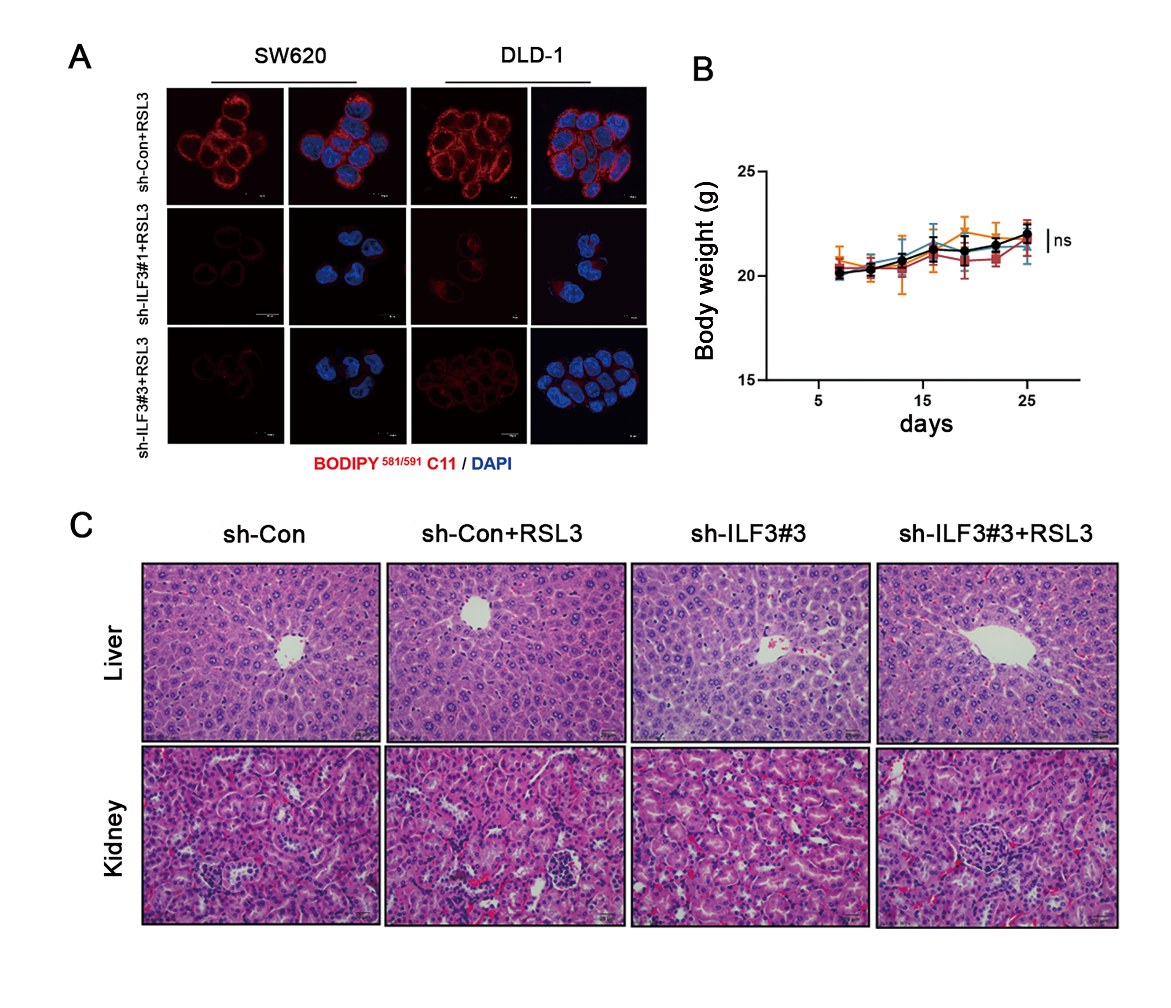


**Fig S2. Knockdown of ILF3 promotes ferroptosis sensitivity in CRC cells. A** Lipid peroxidation levels in sh-Con and sh-ILF3 groups treated with RSL3 (10 μM, 24 h) were detected using a BODIPY (581/591) C11 probe. Scale bar = 10 μm. Red represents reductive status and blue represents DAPI staining. **B** Mouse weight of Xenograft model. **C** HE staining for Liver and kidney of xenograft mouse model.

**Fig S3, related to Fig 4**

**
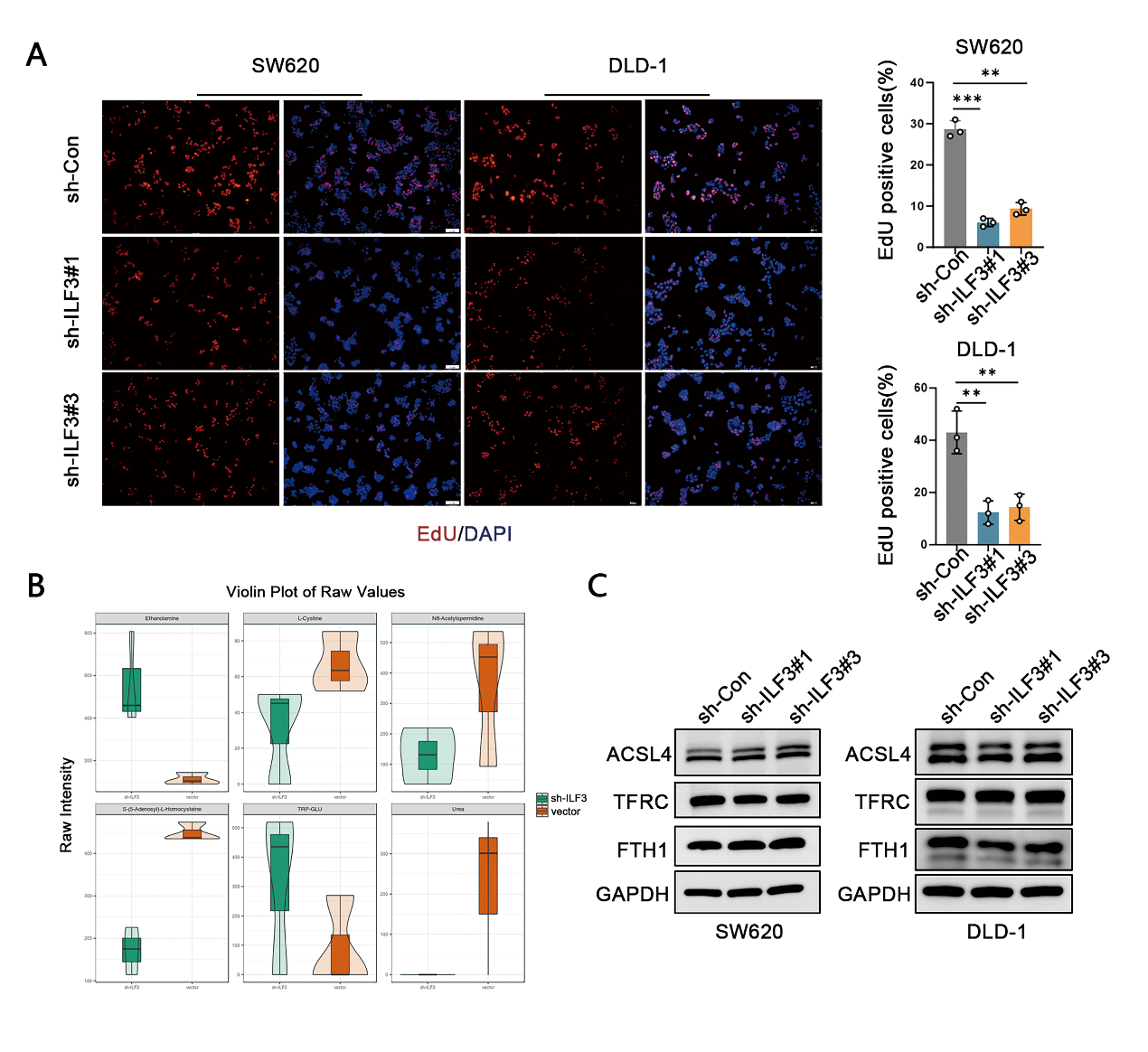
**

**Fig. S3. ILF3 loss inhibits GSH synthesis by suppressing xCT-mediated cystine uptake. A** EdU staining for ILF3 knockdown CRC cells. Red represents EdU staining and blue represents DAPI staining. **B** The differentially expressed metabolites of amino-acid in ILF3 knockdown cells determined by targeted amino-acid metabolomics. **C** The protein expression of ACSL4, TFRC, and FTH1 in ILF3-knockdown SW620 and DLD-1 cells was detected by western blot assay.

**Fig S4, related to Fig 5**


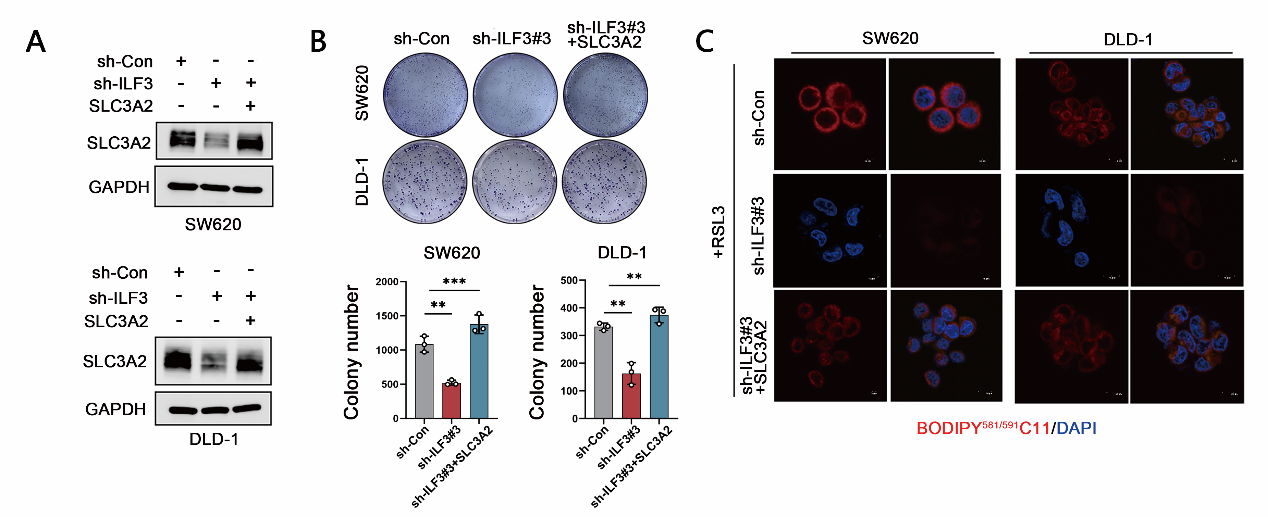


**Fig. S4. ILF3 modulates SLC3A2 mRNA stability by directly binding to the 3’ UTR. A** The protein expression of SLC3A2 in SW620 and DLD-1 cells was detected by western blot assay. **B** Colony formation assay for ILF3 knockdown cells with or without SLC3A2 overexpression. **C** Representative fluorescence images of BODIPY 581/591 C11-labeled lipid peroxidation in ILF3 knockdown cells with or without SLC3A2 overexpression in the presence of RSL3 (10 μM, 24 h). Red represents reductive status and blue represents DAPI staining.

**Fig S5, related to Fig 6**


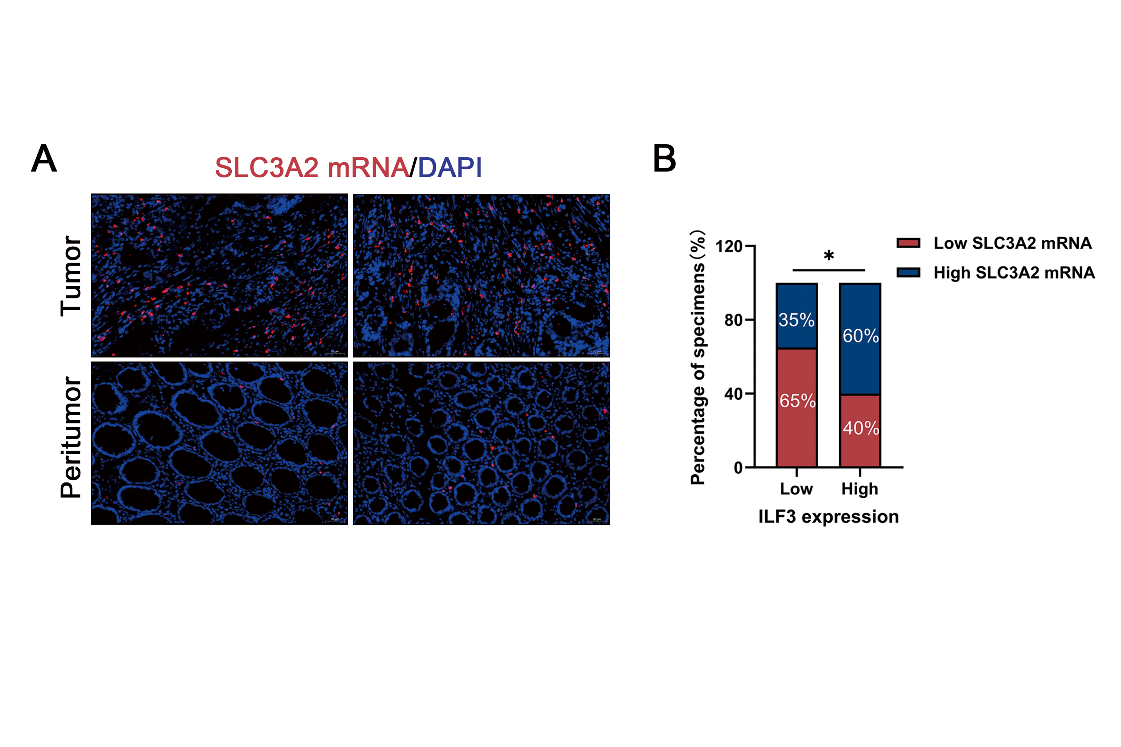


**Fig. S5. ILF3 and SLC3A2 are co-expressed in CRC tissues. A** Representative FISH images of TMA showing SLC3A2 expression in colorectal cancer (CRC) and adjacent normal tissues. Scale bar = 20 μm. **B** Correlation between SLC3A2 mRNA expression and ILF3 protein levels in CRC specimens. *P<0.05

**Fig S6, related to Fig 7**


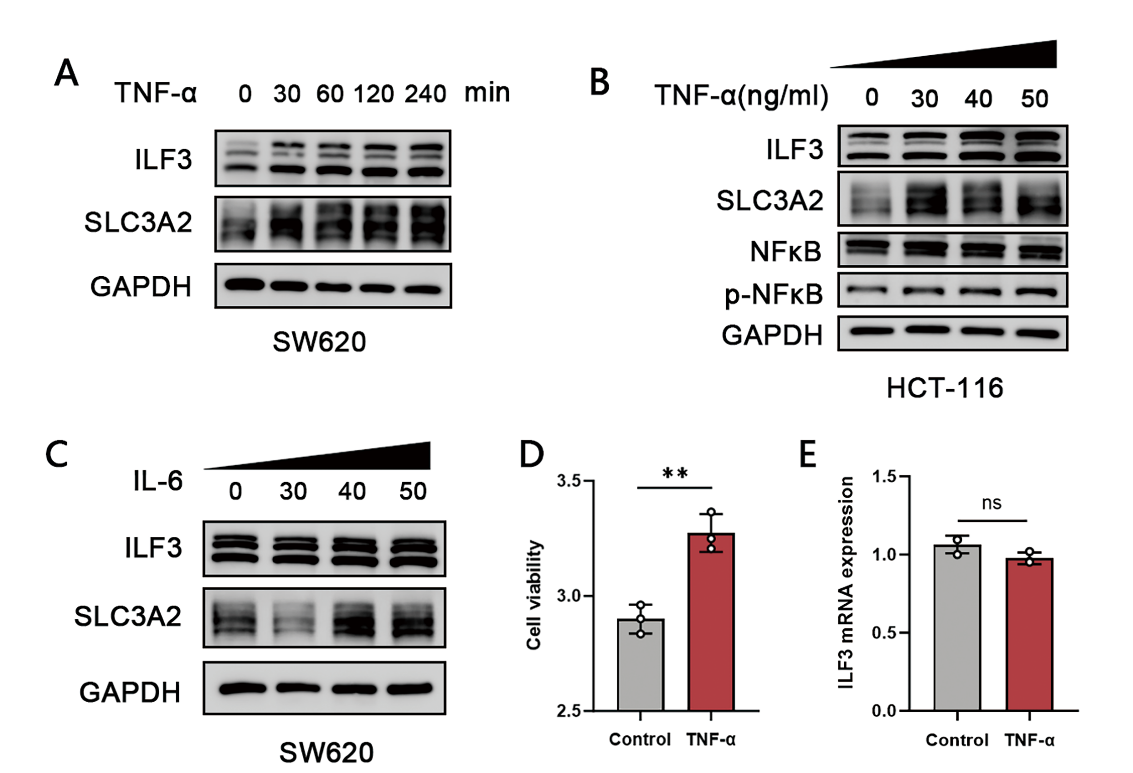


**Fig. S6. TNF-α accelerates cystine uptake and GSH synthesis by upregulating ILF3 expression. A** The protein levels of ILF3 and SLC3A2 in TNF-α-induced (40ng/ml) SW620 cells at different times were detected by western blot assay. **B** HCT-116 cells were treated with different concentrations of TNF-α for 24 h, and protein expression levels of ILF3, SLC3A2, NF-κB p65, and p-NF-κB p65 were analyzed by western blot. **C** The protein levels of ILF3 and SLC3A2 induced by IL-6 at different concentrations in SW620 cells were detected by western blot assay. **D** Cell viability was detected using CCK-8 assay after TNF-α treatment. **E** ILF3 mRNA expression was detected using RT-qPCR after TNF-α treatment.
