# Supplementary material for: ILF3 promotes colorectal cancer cell resistance to ferroptosis by enhancing cysteine uptake and GSH synthesis via stabilizing SLC3A2 mRNA
Source: Cell Death Dis. 2025 Jul 23;16(1):549. doi: 10.1038/s41419-025-07872-x (PMC12284142; doi:10.1038/s41419-025-07872-x)
Supplement: Supplementary file 2 — Supplementary Methods [file 41419_2025_7872_MOESM2_ESM.docx]

**Lentivirus,Plasmids transfection**

SyngenTech(China) constructed ILF3 knockdown lentivirus (pLV-hU6-ILF3 shRNA-hef1a-mNeongreen-P2A-Puro) and SLC3A2 overexpression lentivirus (pLV-hef1a-mScarlet-P2A-neo-WPRE-CMV-SLC3A2-3Xflag) were constructed by Mingsheng Biology (China). Cells were infected for 72 hours with the Enhanced Infection Solution (Polybrene) (SyngenTech) to infect cells for 72 h. Subsequently, ILF3 knockdown cells were screened with 1μg/ml puromycin for two weeks, and SLC3A2 overexpressing cells were screened with 400μg/ml G418 for four weeks.

Myc-ILF3, Flag-Trim17 plasmid was purchased from Mingsheng Biology (China). His-Ub, pCMV-HA-Ub, pCMV-HA-Ub-K6, pCMV-HA-Ub-K11, pCMV-HA-Ub-K27, pCMV-HA-Ub-K29, pCMV-HA-Ub-K33, pCMV-HA-Ub-K48, pCMV-HA-Ub-K63, pCMV-HA-Ub-K29R were constructed by Miaoling Biology (China). The plasmid was transfected into HEK293T cells according to the instructions for Lipofectamine 3000 (Invitrogen, L3000015), and the follow-up experiment was performed 48 hours after transfection.

**RT-qPCR analysis**

Total RNA was extracted using the Animal RNA Extraction Kit (R0027, Beyotime) and reverse transcribed into cDNA using the cDNA First Strand Synthesis Kit (D7168, Beyotime). Relative quantification of RNA levels was performed using SYBR Green qPCR Mix (D7262, Beyotime). CT values were normalized to GAPDH values and the relative expression of mRNA was calculated using the 2 -ΔΔCt method. The primers used are listed in Supplementary Table 2.

**Western blotting analysis**

Cells were washed three times with PBS and lysed on ice with RIPA lysate (R0020, Solarbio) with protease phosphatase inhibitor mixture (P1260, Solarbio) in a 100:1 ratio. Proteins were separated by SDS-PAGE and transferred to PVDF membranes, which were blocked with 5% skimmed milk (2104108, BD) for 1 h. The membranes were incubated with the primary antibody at 4°C overnight. On the next day, the membrane was washed with TBST and the secondary antibody was incubated at room temperature for 1h. After washing the membrane, the antibody signal was detected by ECL.

**Colony formation and cell viability assay**

For colony formation assays, SW620 cells (2000/well) and DLD-1 cells (500/well) were seeded in 6-well plates. When cell clumps were visible with the naked eye, samples were fixed with 4% paraformaldehyde, stained with 0.1% crystal violet and counted using ImageJ software. For the cell viability assay, cells were seeded in 96-well plates (0.5 × 10^4^ cells/well) and grouped for measurement at different time points. 100 μL of culture medium and 10 μL of CCK-8 reagent (IV08-500, Invigentech) were added to each well, and the OD was measured at 450nm after incubation at 37°C for 2 hours.

**EDU incorporation assay**

Cells were inoculated in 96-well plates (0.5*10^4^/well) and incubated for 48 hours, then 100µL of 50µM EdU solution was added and incubated for two hours (C10310-1,RiboBio), PBS buffer was used to wash the cells then 50µL of 4% paraformaldehyde was added and incubated for 30 minutes, 50µL of 2mg/ml glycine was incubated for five minutes to wash and 50µL of osmotic (0.5% Triton X-100 PBS) after ten minutes of incubation, 100ul of 1*Apollo staining reaction solution was added.

**Immunofluorescence analysis**

Inoculate the cells on the cell crawling slides, and allow them to grow to 50%-60% fusion. The cells were then fixed with 4% paraformaldehyde for 10 minutes, permeabilized with 0.1% Triton X-100 (9002-93-1, Macklin) for 10 minutes, and blocked with 0.5% BSA (A8020, Solarbio) for 1 hour at room temperature. The cells were incubated with an appropriate dilution of the primary antibody at 4°C overnight. The next day, the primary antibody was washed away with PBS, and a secondary antibody conjugated to a fluorescent group was added and incubated for 2 hours at room temperature in the dark, followed by three washes with PBS. After washing, the cells were stained with Hoechst (C1027, Beyotime) at 37°C for 10 minutes. In addition, after washing three times with PBS, the slides were sealed with an anti-fluorescence quenching sealing agent (S2100, Solarbio).

**Transmission electron microscopy analysis**

CRC cells were collected for 24 h with or without treatment of RSL3 (HY-100218A,MCE), fixed with 2.5% glutaraldehyde for 24 h, and then with 1% osmium acid for 2 h. The cells were washed three times with 0.1 M pH 7.4 phosphate buffer. The cells were then dehydrated through an ethanol gradient, subsequently embedded in resin, and the mitochondrial structure was observed under a transmission electron microscope.

**GSH/GSSG and MDA assay**

SW620 and DLD-1 cells were inoculated in 100 mm dish and cultured overnight. The cells were then treated with RSL3 (10 μM) for 24 h. PBS was used to wash the cells and harvest the cells. The GSH/GSSG ratio and MDA content in the cells were measured using GSH and GSSG assay kit (S0053, Beyotime) and MDA assay kit (S0131, Beyotime) according to the manufacturer's protocol, respectively.

**Live/dead cell staining of 3D tumor spheroids**

Prepare a 1% agarose gel solution and add 50 μL of the solution to each 96-well plate. Then 1600 cells (200 µL complete medium) are added to each well. On day 7, after cell spheroids had formed, the medium was removed and fresh complete medium containing PBS or 10μM RSL3 was added. After 24 hours of incubation at 37°C with 5% CO2, the 3D tumour spheroids were washed three times with PBS and then stained separately with Calcein/PI according to the manufacturer's instructions (C2015M, Beyotime) for the live/dead cell staining kit. Finally, the cells were imaged using a confocal laser scanning microscope with Z-axis scanning.

**Ni-NTA pulldown**

His protein purification was performed using the His proteintech pulldown kit (BK0055-01, ACE). First, HEK293T cells were washed twice with pre-cooled PBS, digested and centrifuged, and then the cell mass was lysed with 1 ml of pre-cooled lysate, and after centrifugation, the supernatant was removed and 20ul was taken as the input group, followed by 500ul of the remaining supernatant consisting of 500ul of pre-washed 50ul Ni-NTA magnetic beads and incubated for 12 hours on a shaking bed at 4°C. The cells were then washed twice with pre-cooled PBS. Finally, 100ul of 250 mM imidazole was added and incubated for 10 minutes, then centrifuged and 100ul of 2× loading buffer was added to denature the proteins for 10 minutes in a metal bath at 100°C. Finally, the bound proteins were analysed by western blot.
