# Supplementary material for: ILF3 promotes colorectal cancer cell resistance to ferroptosis by enhancing cysteine uptake and GSH synthesis via stabilizing SLC3A2 mRNA
Source: Cell Death Dis. 2025 Jul 23;16(1):549. doi: 10.1038/s41419-025-07872-x (PMC12284142; doi:10.1038/s41419-025-07872-x)
Supplement: Supplementary file 3 — Supplementary tables [file 41419_2025_7872_MOESM3_ESM.docx]

| **Supplementary Table 1.** Relationship between ILF3 expression and the clinicopathological features in CRC. | | | | |
| --- | --- | --- | --- | --- |
| Characteristic | No. of cases | Strongly positive cases (%) | **χ^2^** | *P* value |
| **Gender** |  |  | 0.015 | 0.902 |
| Male | 40 | 30(75.0%) |  |  |
| Female | 42 | 31(73.8%) |  |  |
| **Age (years old)** |  |  | 0.019 | 0.892 |
| ≤65 | 44 | 33 (75.0%) |  |  |
| ＞65 | 38 | 28(73.7%) |  |  |
| **Tumor size (cm)** |  |  | 0.792 | 0.373 |
| ≤5 | 43 | 33(76.7%) |  |  |
| ＞5 | 34 | 23 (67.6%) |  |  |
| **Differentiation** |  |  | 3.921 | 0.048* |
| Well and moderately diff. | 72 | 51 (70.8%) |  |  |
| Poorly diff. | 10 | 10 (100.0%) |  |  |
| **TNM clinical stage** |  |  | 2.746 | 0.097 |
| I-II | 50 | 34(68.0%) |  |  |
| III-IV | 32 | 27 (84.4%) |  |  |
| **pT** |  |  | 3.48 | 0.062 |
| T1-2 | 9 | 9 (100.0%) |  |  |
| T3-4 | 73 | 52 (71.2%) |  |  |
| **Lymph node metastasis** |  |  | 1.986 | 0.159 |
| + | 30 | 25(83.3%) |  |  |
| - | 52 | 36 (69.2%) |  |  |
| [**Distant**](javascript:;) [**metastasis**](javascript:;) |  |  | 1.833 | 0.176 |
| + | 5 | 5(100.0%) |  |  |
| - | 77 | 56(72.7%) |  |  |

* *p*<0.05.

**Supplementary Table 2. The primer sequence of indicated gene.**

| **No.** | **Primer Name** | **Sequence (5'to3')** |
| --- | --- | --- |
| 1 | GNL3-F | ATGACCTGCCATAAGCGGTAT |
| 2 | GNL3-R | CTTAAAGGGAGCACTGTTTGGA |
| 3 | HNRNPK-F | CAATGGTGAATTTGGTAAACGCC |
| 4 | HNRNPK-R | GTAGTCTGTACGGAGAGCCTTA |
| 5 | ILF3-F | AGCATTCTTCCGTTTATCCAACA |
| 6 | ILF3-R | GCTCGTCTATCCAGTCGGAC |
| 7 | LARP7-F | CGGTCACGAGTTAAACAGGTG |
| 8 | LARP7-R | GCCTTCCAAATCAAGCTCTACAA |
| 9 | SYNE1-F | ACCTCCAATGGTGGTGGAC |
| 10 | SYNE1-R | CGTGCCAATGTTAGCCACA |
| 11 | TAF15-F | GATTCTGGAAGTTACGGTCAGTC |
| 12 | TAF15-R | AGCTTTGTGATGCTTGTCCATAG |
| 13 | ZFP36-F | GACTGAGCTATGTCGGACCTT |
| 14 | ZFP36-R | GAGTTCCGTCTTGTATTTGGGG |
| 15 | YWHAZ-F | TGTAGGAGCCCGTAGGTCATC |
| 16 | YWHAZ-R | GTGAAGCATTGGGGATCAAGA |
| 17 | MBNL1-F | GCTGTTAGTGTCACACCAATTCG |
| 18 | MBNL1-R | AGGCGATTACTCGTCCATTTTC |
| 19 | MBNL2-F | TCAAAGAGGAACATGCTCACG |
| 20 | MBNL2-R | AACGGCCCTTTAGGGAATCAA |
