# Supplementary material for: ILF3 promotes colorectal cancer cell resistance to ferroptosis by enhancing cysteine uptake and GSH synthesis via stabilizing SLC3A2 mRNA
Source: Cell Death Dis. 2025 Jul 23;16(1):549. doi: 10.1038/s41419-025-07872-x (PMC12284142; doi:10.1038/s41419-025-07872-x)

Fig 1F

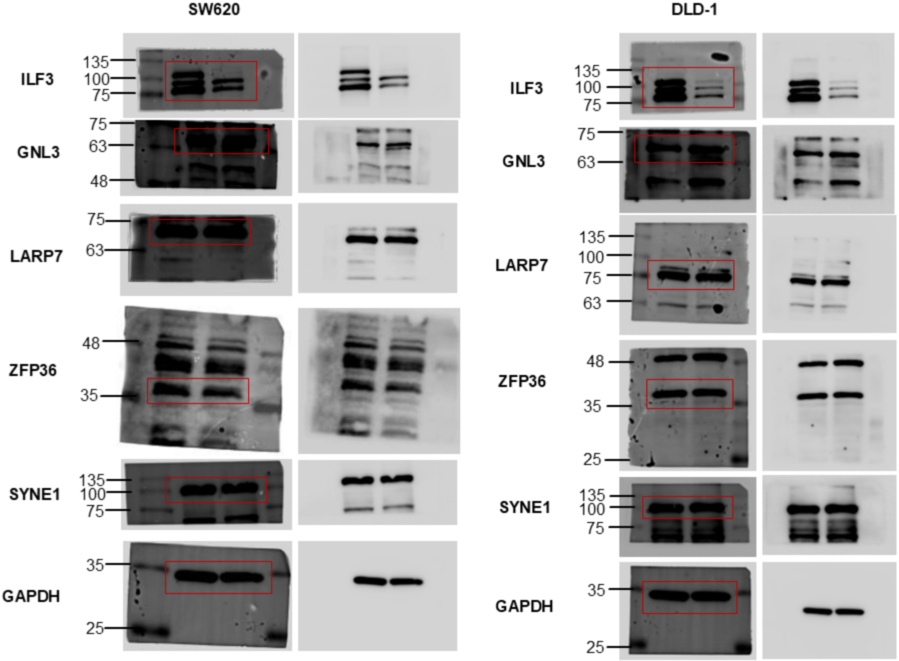
SW620
DLD-1
ILF3
GNL3
LARP7
ZFP36
SYNE1
GAPDH
135
100
75
63
48
35
25

Fig 1G

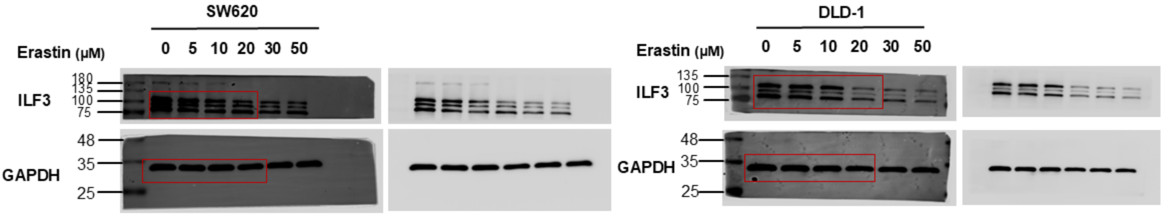
SW620
Erastin (μM)
0 5 10 20 30 50
ILF3
180
135
100
75
GAPDH
48
35
25
DLD-1
Erastin (μM)
0 5 10 20 30 50
ILF3
135
100
75
GAPDH
48
35
25

Fig 1H

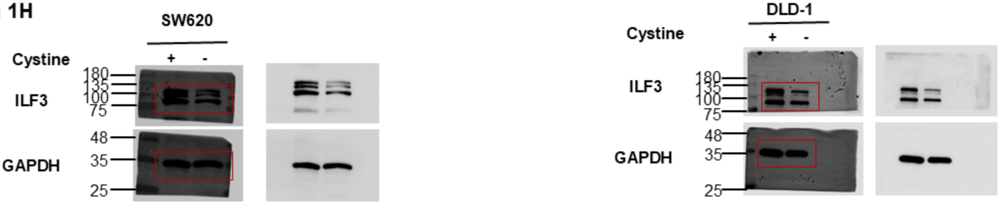
SW620
Cystine
+ -
ILF3
180
135
100
75
GAPDH
48
35
25
DLD-1
Cystine
+ -
ILF3
180
135
100
75
GAPDH
48
35
25

Fig 2A

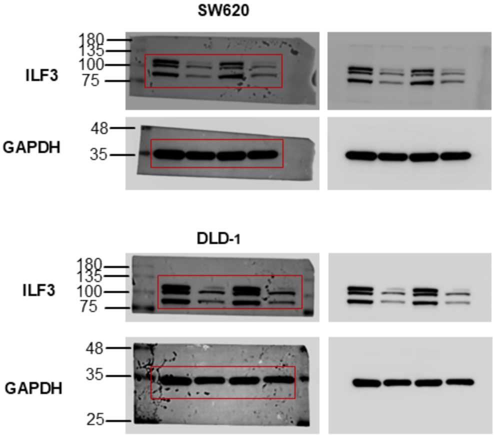
SW620
ILF3
180
135
100
75
GAPDH
48
35
DLD-1
ILF3
180
135
100
75
GAPDH
48
35
25

Fig 3D

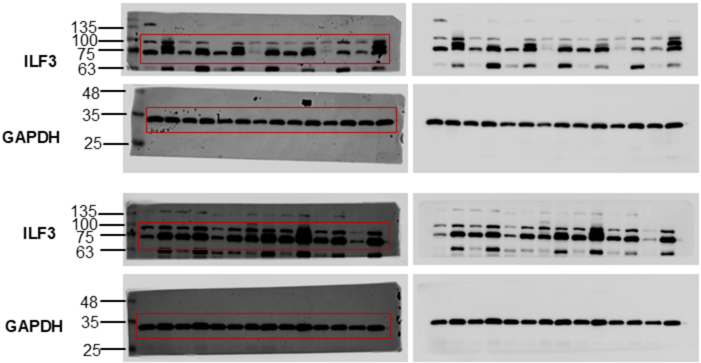
ILF3
135
100
75
63
GAPDH
48
35
25
ILF3
135
100
75
63
GAPDH
48
35
25

Fig 4G

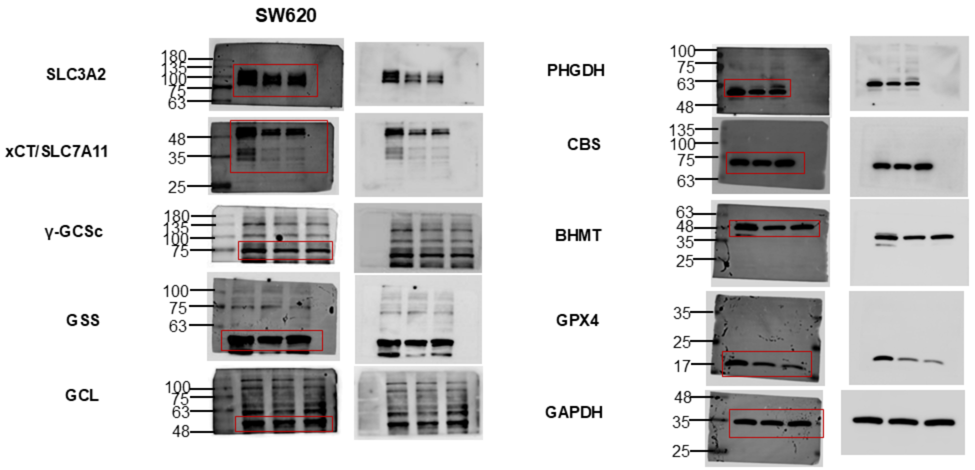
SW620
SLC3A2
180
135
100
75
63
xCT/SLC7A11
48
35
25
γ-GCSc
180
135
100
75
GSS
100
75
63
GCL
100
75
63
48
PHGDH
100
75
63
48
CBS
135
100
75
63
BHMT
63
48
35
25
GPX4
35
25
17
GAPDH
48
35
25

Fig 4G

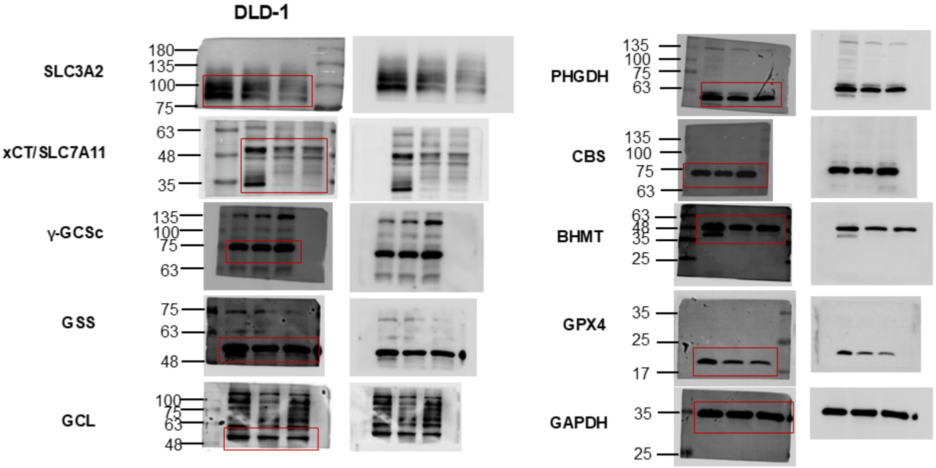
DLD-1
SLC3A2
180
135
100
75
xCT/SLC7A11
63
48
35
γ-GCSc
135
100
75
63
GSS
75
63
48
GCL
100
75
63
48
PHGDH
135
100
75
63
CBS
135
100
75
63
BHMT
63
48
35
25
GPX4
35
25
17
GAPDH
35
25

Fig 6l

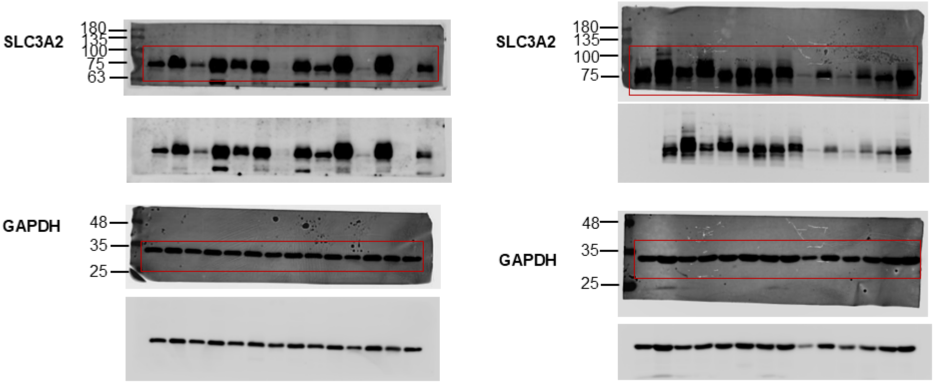
SLC3A2
180
135
100
75
63
GAPDH
48
35
25
SLC3A2
180
135
100
75
GAPDH
48
35
25

Fig 7A

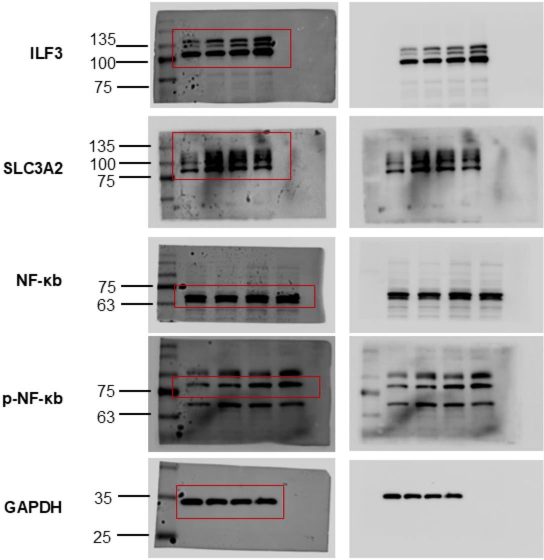
ILF3
135
100
75
SLC3A2
135
100
75
NF-κb
75
63
p-NF-κb
75
63
GAPDH
35
25

Fig 7G

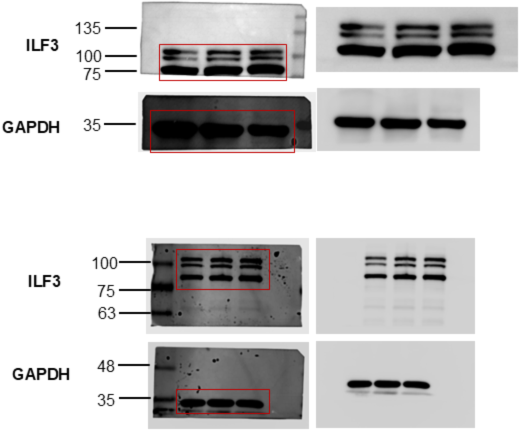
ILF3
135
100
75
GAPDH
35
ILF3
100
75
63
GAPDH
48
35

Fig 7H

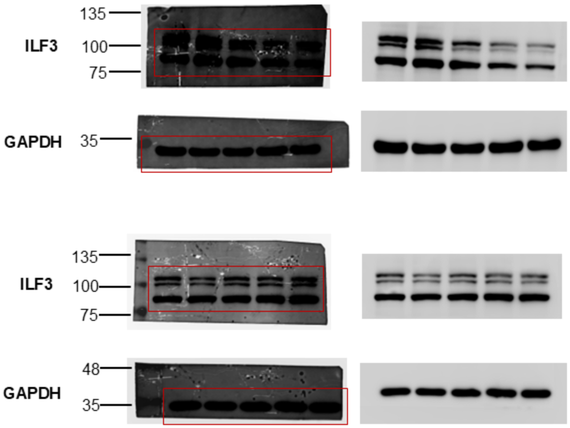
ILF3
135
100
75
GAPDH
35
ILF3
135
100
75
GAPDH
48
35

Fig 7I

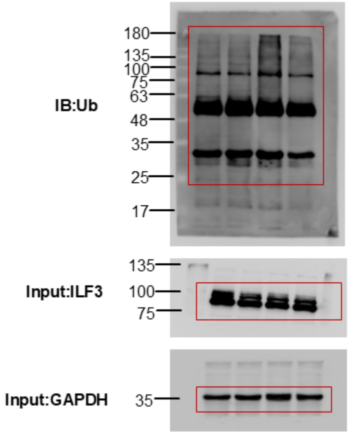
IB:Ub
180
135
100
75
63
48
35
25
17
Input:ILF3
135
100
75
Input:GAPDH
35

Fig 7J

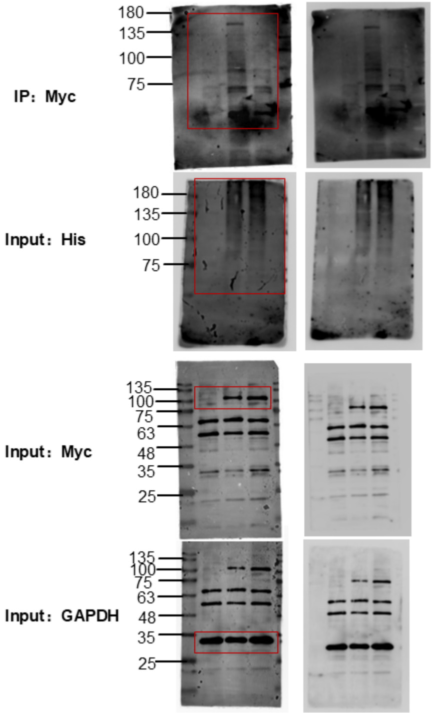
IP：Myc
180
135
100
75
Input：His
180
135
100
75
Input：Myc
135
100
75
63
48
35
25
Input：GAPDH
135
100
75
63
48
35
25

Fig 7K

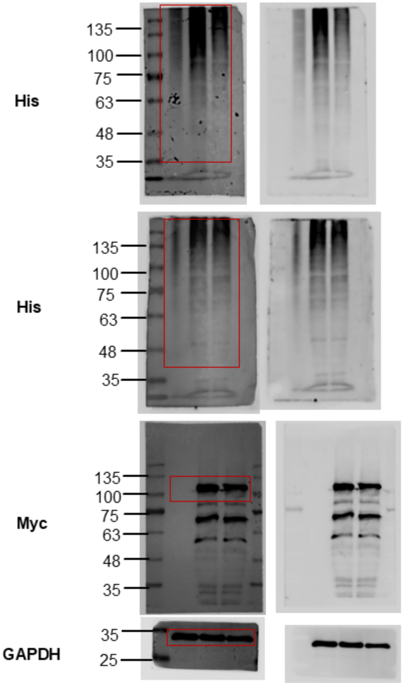
His
135
100
75
63
48
35
His
135
100
75
63
48
35
Myc
135
100
75
63
48
35
GAPDH
35
25

Fig 8D

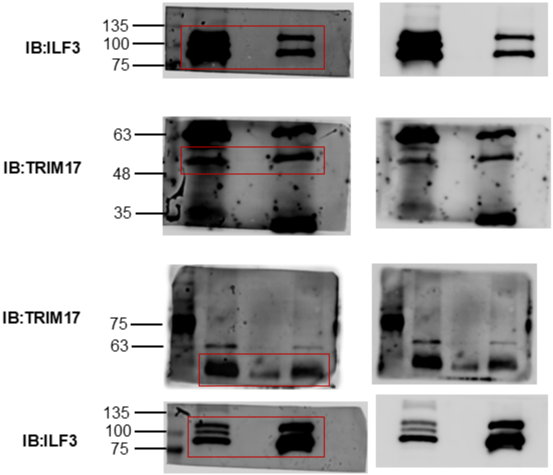
IB:ILF3
135
100
75
IB:TRIM17
63
48
35
IB:TRIM17
75
63
IB:ILF3
135
100
75

Fig 8E

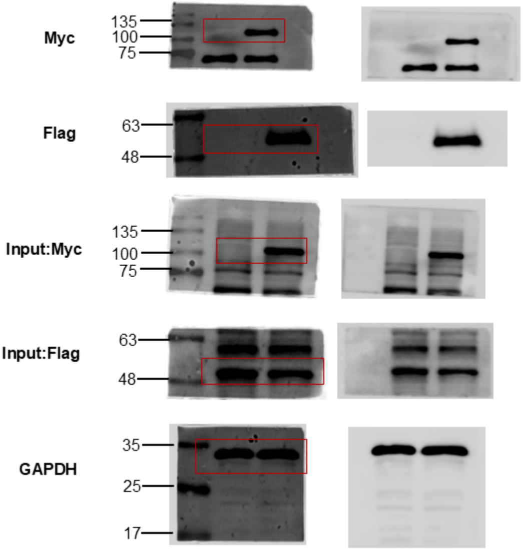
Myc
135
100
75
Flag
63
48
Input:Myc
135
100
75
Input:Flag
63
48
GAPDH
35
25
17

Fig 8F

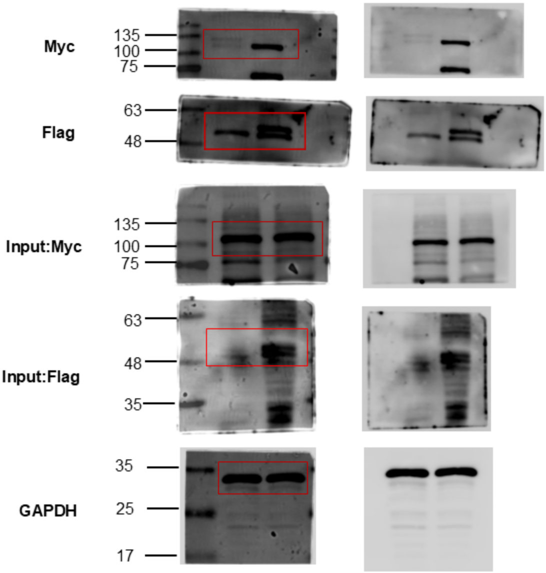
Myc
135
100
75
Flag
63
48
Input:Myc
135
100
75
Input:Flag
63
48
35
GAPDH
35
25
17

Fig 8G

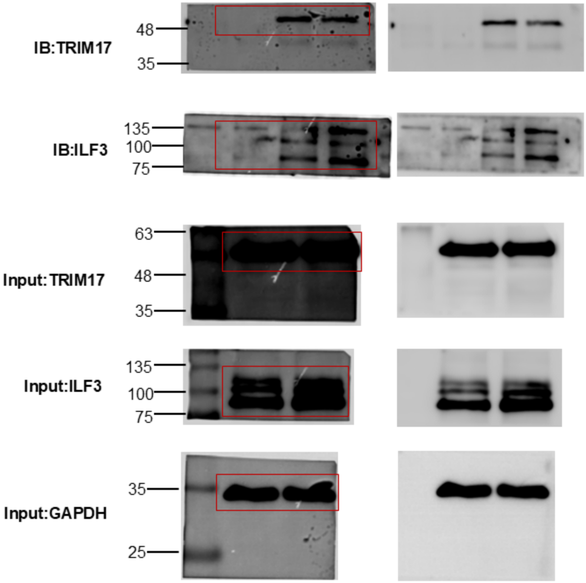
IB:TRIM17
48
35
IB:ILF3
135
100
75
Input:TRIM17
63
48
35
Input:ILF3
135
100
75
Input:GAPDH
35
25

Fig 8H

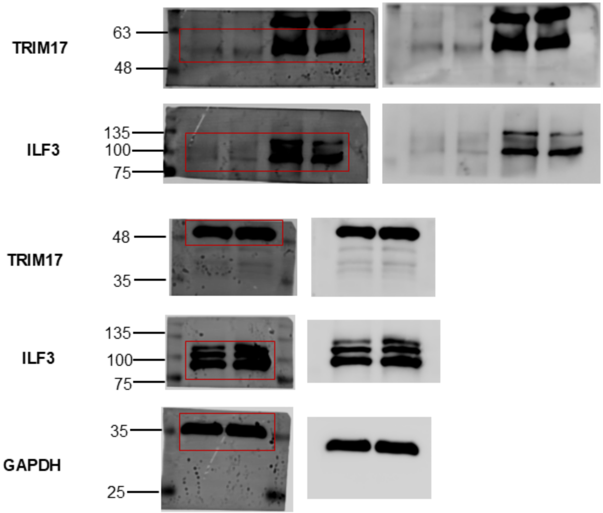
TRIM17
63
48
ILF3
135
100
75
TRIM17
48
35
ILF3
135
100
75
GAPDH
35
25

Fig 8I

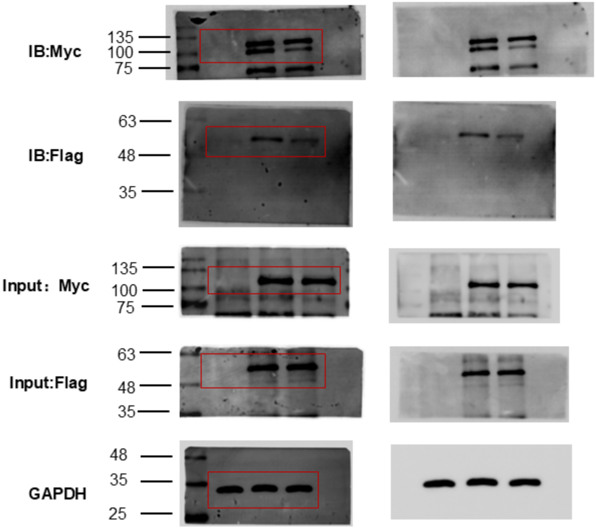
IB:Myc
135
100
75
IB:Flag
63
48
35
Input：Myc
135
100
75
Input:Flag
63
48
35
GAPDH
48
35
25

Fig 8J

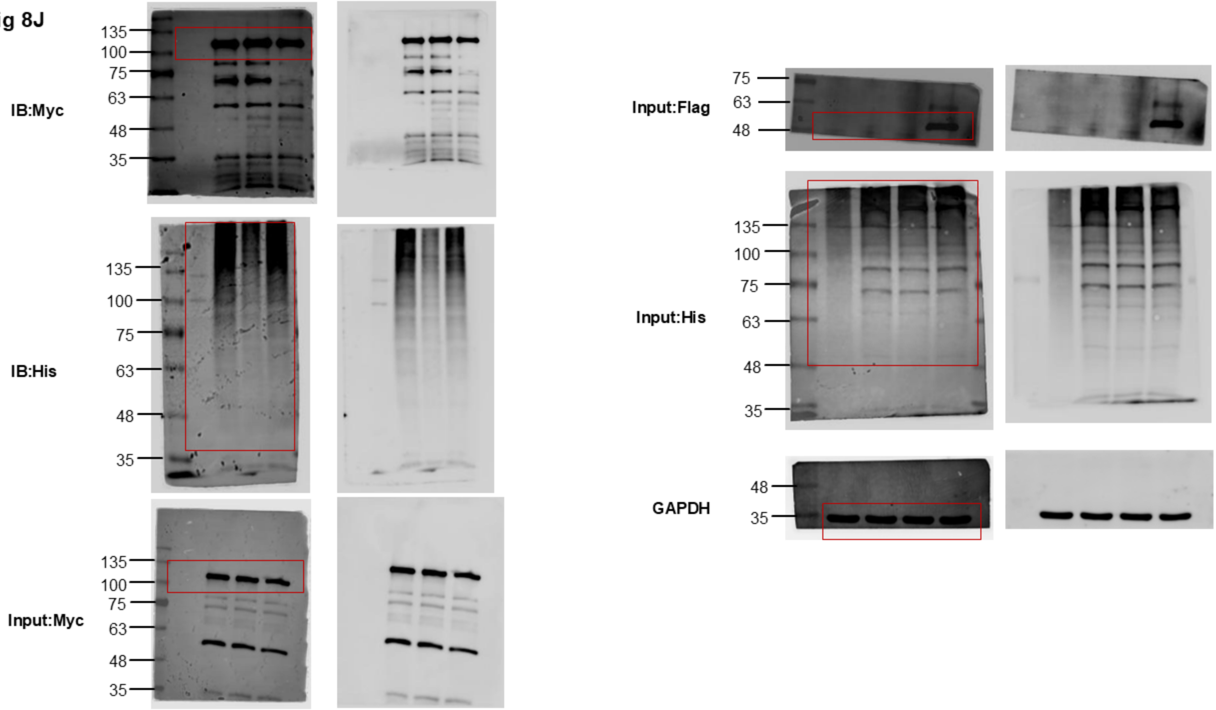
IB:Myc
135
100
75
63
48
35
IB:His
135
100
75
63
48
35
Input:Myc
135
100
75
63
48
35
Input:Flag
75
63
48
Input:His
135
100
75
63
48
35
GAPDH
48
35

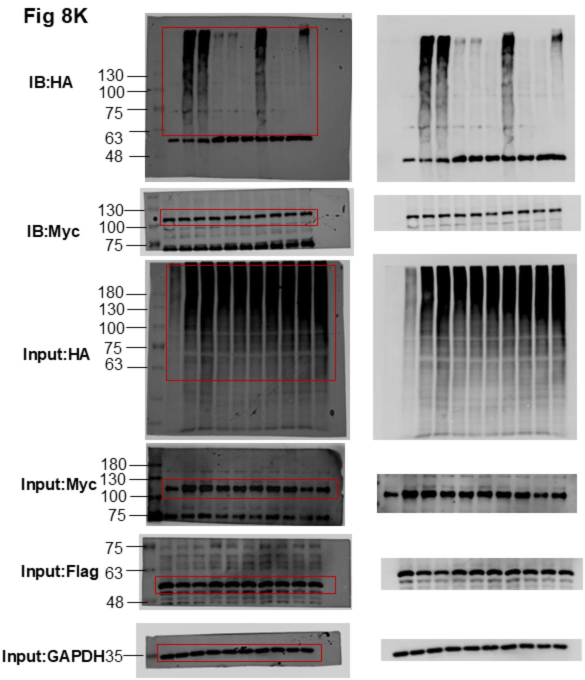
Fig 8K
IB:HA
130
100
75
63
48
IB:Myc
130
100
75
Input:HA
180
130
100
75
63
Input:Myc
180
130
100
75
Input:Flag
75
63
48
Input:GAPDH
35

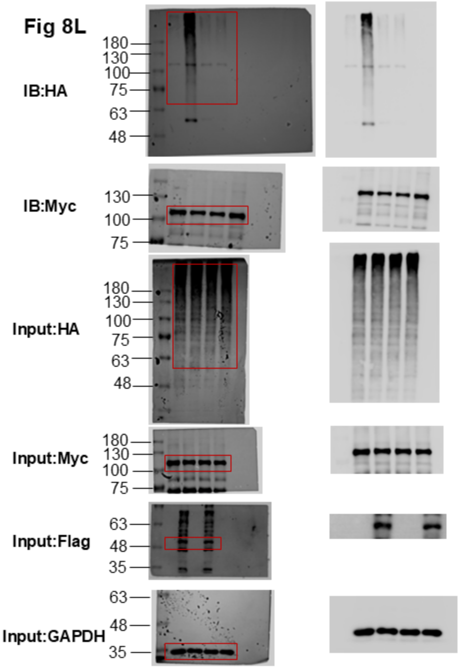
Fig 8L
IB:HA
180
130
100
75
63
48
IB:Myc
130
100
75
Input:HA
180
130
100
75
63
48
Input:Myc
180
130
100
75
Input:Flag
63
48
35
Input:GAPDH
63
48
35

Fig S1A

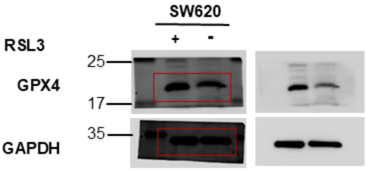

Fig S1B

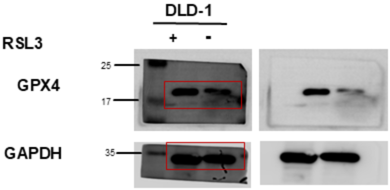

Fig S3C

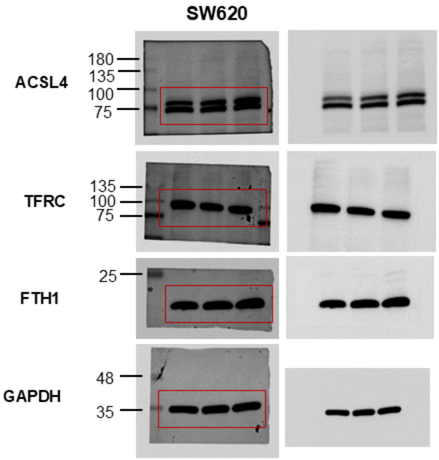

Fig S3C

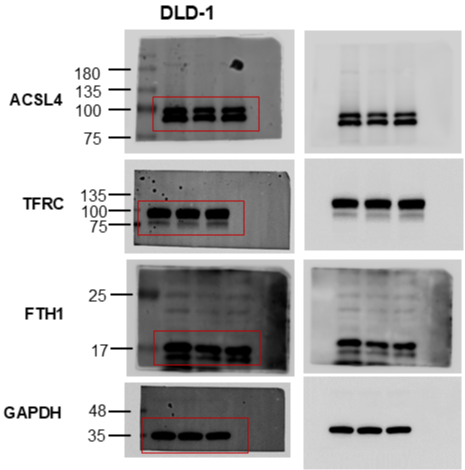

Fig S4A

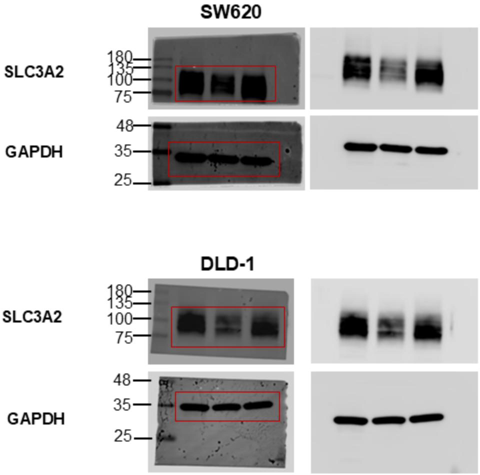

Fig S6A

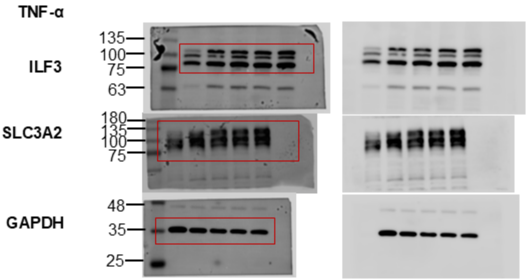

Fig S6B

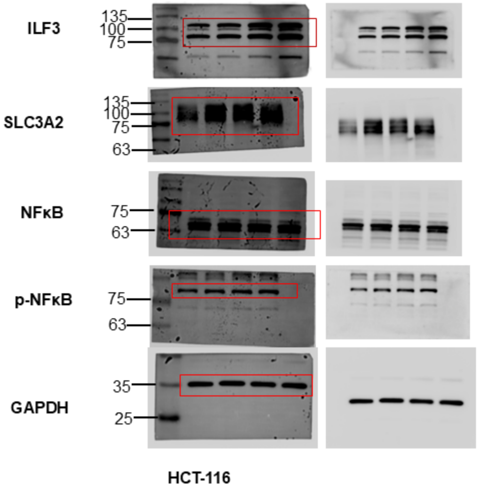

Fig S6C

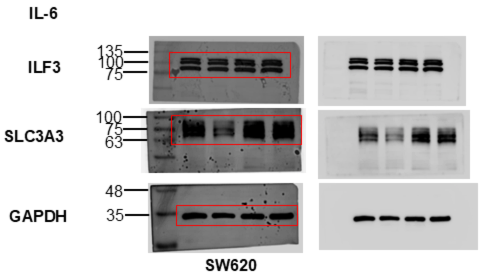

Supplement: Supplementary file 4 — Uncropped original western blots [file 41419_2025_7872_MOESM4_ESM.pdf]
